# Plasma-based multi-reforming for Gas-To-Liquid: tuning the plasma chemistry towards methanol

**DOI:** 10.1038/s41598-018-34359-x

**Published:** 2018-10-29

**Authors:** Ramses Snoeckx, Weizong Wang, Xuming Zhang, Min Suk Cha, Annemie Bogaerts

**Affiliations:** 1 King Abdullah University of Science and Technology (KAUST), Clean Combustion Research Center (CCRC), Physical Science and Engineering Division (PSE), Thuwal, 23955 Saudi Arabia; 2 Research group PLASMANT, Department of Chemistry, University of Antwerp, Universiteitsplein 1, BE-2610 Antwerp, Belgium; 3 Present Address: College of Environmental Science and Engineering, Zhejiang Gongshang University, Xiasha High Education District, Hangzhou, Zhejiang Province China

## Abstract

Because of its unique properties, plasma technology has gained much prominence in the microelectronics industry. Recently, environmental and energy applications of plasmas have gained a lot of attention. In this area, the focus is on converting CO_2_ and reforming hydrocarbons, with the goal of developing an efficient single-step ‘gas-to-liquid’ (GTL) process. Here we show that applying tri-reforming principles to plasma—further called ‘plasma-based multi-reforming’—allows us to better control the plasma chemistry and thus the formed products. To demonstrate this, we used chemical kinetics calculations supported by experiments and reveal that better control of the plasma chemistry can be achieved by adding O_2_ or H_2_O to a mixture containing CH_4_ and CO_2_ (diluted in N_2_). Moreover, by adding O_2_ and H_2_O simultaneously, we can tune the plasma chemistry even further, improving the conversions, thermal efficiency and methanol yield. Unlike thermocatalytic reforming, plasma-based reforming is capable of producing methanol in a single step; and compared with traditional plasma-based dry reforming, plasma-based multi-reforming increases the methanol yield by more than seven times and the thermal efficiency by 49%, as revealed by our model calculations. Thus, we believe that by using plasma-based multi-reforming, ‘gas-to-liquid’ conversion may be made efficient and scalable.

## Introduction

The world needs an easily scalable gas reforming technology more than ever. From an environmental perspective, we need a technology that can efficiently convert CO_2_—together with a co-reactant—into value-added chemicals and fuels^1–6^. From an energetic and economic perspective, we want a technology that can transform gaseous hydrocarbons (mainly methane) into liquids; this is known as the ‘gas-to-liquid’ (GTL) process^7^. Interest in GTL began in the early 20^th^ century; yet, despite some first commercial GTL plants, we still do not have an efficient scalable GTL process to convert gaseous hydrocarbons, that are currently flared in the (petro)chemical industry. An efficient GTL process would make it possible to liquefy both natural gas and methanated CO_2_, and would enable us to efficiently utilize the growing feedstocks of biogas and landfill gas. In contrast to GTL, interest in converting CO_2_ is more recent, still both GTL and CO_2_ conversion technologies share a common goal; both technologies aim to convert gases into value-added chemicals and fuels.

In 2004 Song *et al*.^8^ introduced a novel concept called tri-reforming of methane. The proposed single reactor process was a combination of dry reforming of methane (DRM), steam methane reforming (SMR) and partial oxidation of methane (POX). By this process, syngas with industrially desired H_2_/CO ratios (1.5–2.0) can be produced and the DRM coking issue can be eliminated^8,9^. Nevertheless, this innovative approach still relies on several energy intensive post-processing steps in order to transform the syngas into desired liquid products^10^. This issue makes a single-step GTL process that we present here more beneficial. It is exactly in this area that non-thermal plasma technology could excel^1,11,12^. A key feature of plasma technology is that it can activate gas molecules at room temperature through electron impact reactions, rather than through heat. Several plasma reactors have already been studied for a variety of plasma-based reforming processes, including pure CO_2_ splitting, CO_2_ hydrogenation, artificial photosynthesis, methane reforming, DRM, SMR and POX^1,11–13^. However, obtaining a high selectivity and yield of the desired chemicals and fuels proves to be challenging.

Recently, adding O_2_ to the mixture has gained some attention^14–23^, but adding H_2_O to plasma-based DRM has not, given that, to our knowledge, only two papers on this topic exist in the literature^24,25^. Here we show that introducing the principles of tri-reforming in a plasma reactor significantly enhances the conversions, thermal efficiency and methanol yield. This is the first fundamental investigation on the benefits of—what we will further call—‘plasma-based multi-reforming’.

## Combining Experiments and Chemical Kinetics Calculations

To investigate the possibilities of plasma-based multi-reforming, we performed chemical kinetics calculations supported by experiments. The reactor used for the experiments^26–28^ and the chemistry set^29^ (including its experimental validation) used for the calculations were presented previously. A detailed sensitivity analysis on this chemistry set was presented by Wang *et al*.^30^ (detailed information on both the model and experiments can be found in the respective references as well as the Supplementary Information).

The experimental setup consists of a temperature-controlled coaxial DBD reactor with a plasma reactor volume of 3.31 cm^3^, a reactant feeding system, a high-voltage power supply, and an analytical system. The experiments were performed for a fixed temperature of 673 K (to prevent condensation of the liquid products) and a fixed pressure of 1 atm. The Specific Energy Input (SEI) is defined as the plasma power divided by the total gas flow rate, while the energy for heating the gas is taken into account separately when calculating the thermal efficiency (see equation S22 in the Supplementary Information).

The model used in this work to predict the plasma chemistry is a zero-dimensional (0D) chemical kinetics model, called ZDPlaskin^31^. In this model, the time evolution of the species densities is calculated by balance equations, taking into account the various production and loss terms by chemical reactions. The temperature-dependent rate coefficients of the heavy particle reactions (i.e., atoms, molecules, radicals, ions, and excited species) are assumed to be constant, in accordance to the findings reported in the literature and were adopted for the temperature of 673 K, used in the experiments. The rate coefficients for the electron impact reactions are calculated with a Boltzmann solver, BOLSIG+^32^, which is integrated into ZDPlaskin. A more detailed description of ZDPlaskin is provided by Pancheshnyi *et al*.^31^.

Here, we first validated the general model in more detail, based on a series of specific experiments with the addition of 2 up to 8% O_2_ or H_2_O, of which the results can be found in the Supplementary Information. We then further explore the plasma-based multi-reforming process relying on extensive model calculations only, in which we extrapolate the validated range up to 32% O_2_ or H_2_O added, as well as for the combined addition of O_2_ and H_2_O. Hence, these results should be qualitatively evaluated (rather than quantitatively) as an indication of the possibilities the addition of specific compounds has on tuning the chemistry. Especially, to our knowledge this is the first study that focuses not just on using a model for analysing the plasma reforming chemistry, but actually using the plasma chemistry to specifically tailor the production of chemicals based on the insights obtained from such previous combined modelling and experimental studies. As a result it is a—much needed—way more fundamental change to looking at plasma reforming processes.

In the Supplementary Information, we report very detailed information about the experiments and the model used, including detailed experimental and modelling results, and equations to calculate conversions, yields, selectivities and thermal efficiency. Also in the Supplementary Information, we report the comparison between the experimental and modelling results. We will limit the discussion (sections 3, 4 and 5 below) to modelling results only to highlight the potential impact of plasma-based multi-reforming in the field of gas reforming, thus stressing the importance of further research in this field.

## Controlling the Chemistry by Moving to Plasma-Based Multi-Reforming

**Figure 1:**
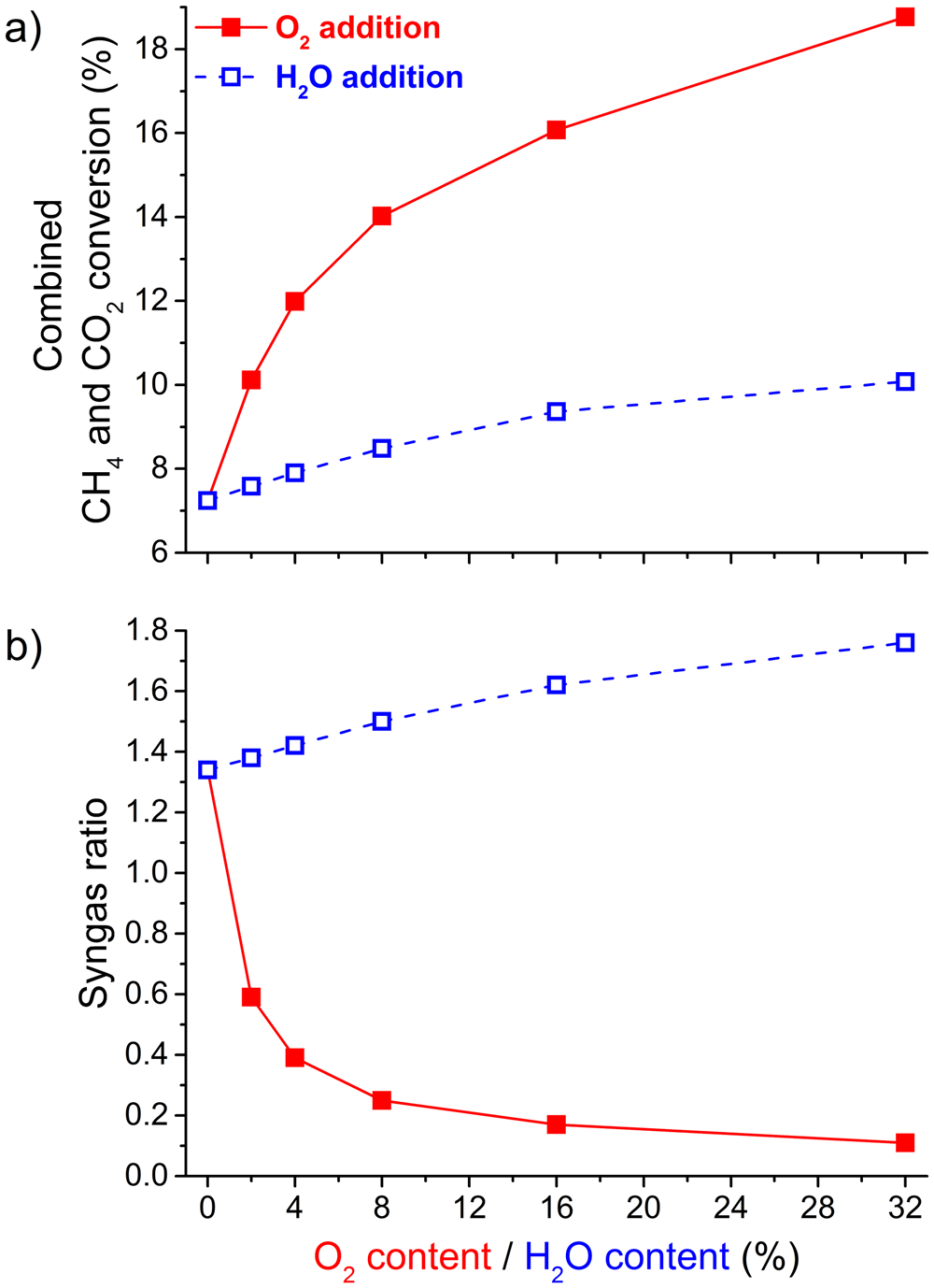
Calculated values of the combined CH_4_ and CO_2_ conversion (**a**); and the syngas ratio (H_2_/CO) (**b**) as a function of O_2_ or H_2_O content, for a modelled DBD operating at an SEI of 3 kJ/L, with a mixture of 10% CH_4_ and 10% CO_2_ diluted in N_2_.

**Figure 2:**
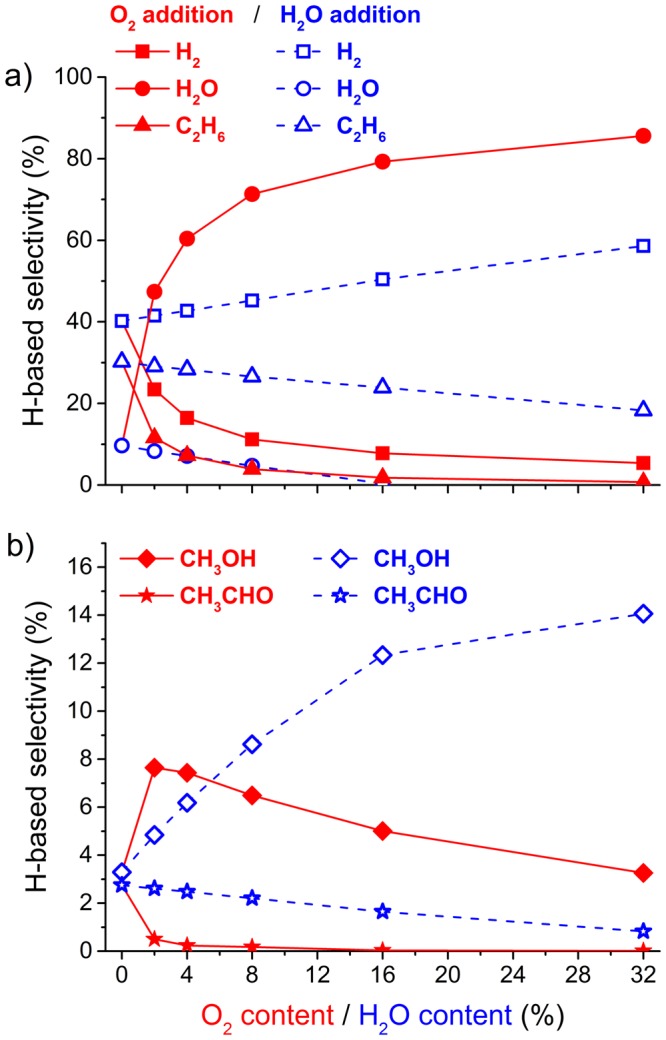
Calculated values of the H-based selectivity towards H_2_, H_2_O, C_2_H_6_ (**a**), and CH_3_OH and CH_3_CHO (**b**) as a function of O_2_ or H_2_O content, for a modelled DBD operating at an SEI of 3 kJ/L, with a mixture of 10% CH_4_ and 10% CO_2_ diluted in N_2_.

A central issue in plasma-based reforming processes is to enhance the selectivity to liquid products over the selectivity to gaseous products (mainly syngas)^1,12,33^. As a baseline case, we modelled a DBD operating at an SEI of 3 kJ/L, with a DRM mixture of 10% CH_4_ and 10% CO_2_ diluted in 80% N_2_. This yielded a H-based selectivity towards methanol of only 3.29%, while the main products were H_2_ (40.2%), C_2_H_6_ (30.2%) and H_2_O (9.65%), as shown in Figs 1 and 2. (More details are provided in the Supplementary Information). We focus on the H-based selectivity, not the C-based selectivity (which can be found in the Supplementary Information), because the hydrogen atoms are the most valuable species for this application. These selectivities resulted in a syngas ratio of 1.34 for the standard plasma-based DRM baseline case. To explore the specific effects of O_2_ and H_2_O through plasma-based multi-reforming, we added either O_2_ or H_2_O to the mixture. In both cases, we kept the CH_4_ and CO_2_ concentrations fixed at 10%, with the remainder being N_2_.

At first, adding O_2_ seemed to be more beneficial than adding H_2_O, as 2% of O_2_ was sufficient to markedly increase, the combined conversion of CH_4_ and CO_2_ (Fig. 1a) and methanol selectivity (Fig. 2b). However, we observed that the syngas ratio decreased (Fig. 1b), and the H_2_O selectivity increased (Fig. 2a) as soon as O_2_ was added, which leads us to conclude the opposite. O_2_—and especially its electron impact dissociation product O—is a strong oxidant and thus very effective at converting CH_4_. This explains why the combined conversion rapidly increased. Unfortunately, O_2_ mainly converted CH_4_ into H_2_O and CO/CO_2_. When 2 to 8% of O_2_ was added, 50 to 70% of the hydrogen atoms from CH_4_ were lost to H_2_O. This is a huge loss because the hydrogen atoms are the most valuable species. Preferably we aspire them to form methanol (CH_3_OH) or hydrogen gas (H_2_), definitely not water (H_2_O).

**Figure 3:**
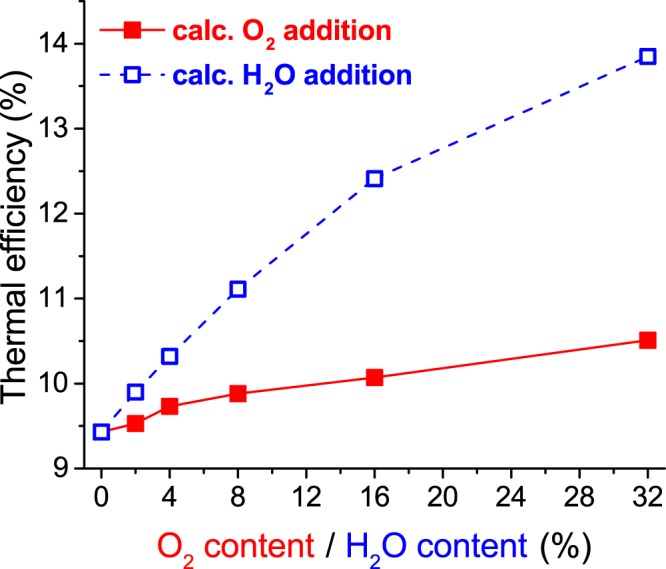
Calculated values of the thermal efficiency as a function of O_2_ or H_2_O content, for a modelled DBD operating at an SEI of 3 kJ/L, with a mixture of 10% CH_4_ and 10% CO_2_ diluted in N_2_.

When H_2_O was added to the mixture, instead of O_2_, the combined conversion of CH_4_ and CO_2_ increased slowly but steadily (Fig. 1a), and the syngas ratio also increased slowly but steadily (Fig. 1b). These results are contrary to the fact that adding H_2_O is detrimental to the conversion process in a DBD for a mixture containing only CO_2_^34^. The most interesting result came from the selectivity. Contrary to O_2_, when H_2_O was added, the selectivity of H_2_O decreased whereas the selectivity of H_2_ and that of CH_3_OH increased (Fig. 2a,b). The selectivity of the other main products, such as CH_3_CHO and C_2_H_6_, decreased as the H_2_O content increased. When the H_2_O fraction reached 32%, a selectivity of 14.1% was obtained for methanol, 58.6% for H_2_, 18.3% for C_2_H_6_ and there was no more selectivity to H_2_O (Fig. 2); in fact, 0.21% of the initial H_2_O was effectively converted. This change in H-based selectivity from H_2_O to more valuable products also led to a higher thermal efficiency when H_2_O was added compared to when O_2_ was added (Fig. 3).

The methanol selectivity appears to saturate when the H_2_O content reaches 32% (Fig. 2b). This indicates that we are near a point of chemical equilibrium for these specific conditions of the plasma. By adding H_2_O to the mixture, we shifted the chemistry from water production to methanol production. Details are provided in Fig. 2 (for selectivities) and in Supplementary Fig. S3 (for yields). At one point, between 16 and 32% water, so much water is added that the chemistry undergoes another change and effectively starts to convert part of the water present in the mixture (see details in the Supplementary Information). Despite the thermodynamic non-equilibrium character of the plasma and its influence on reactive species generation, it appears that the overall reaction chemistry is still following Le Chateliers’ principle. From a chemist’s perspective, it is reasonable to believe that this is partially the case, together with the physical changes that influence the chemistry.

When 32% of water was added, the effective conversion of CH_4_ increased by 44% (from 1.01 to 1.45%), that of CO_2_ increased by 27% (from 0.44 to 0.56%), and the syngas ratio increased by 31% (from 1.34 to 1.76). At the same time, the (H-based) methanol selectivity increased by more than a factor 4 (from 3.29 to 14.1%), and the yield increased by more than a factor 6 (from 0.033 to 0.219%). Finally, this led to a 47% increase in the thermal efficiency (from 9.43 to 13.85%).

Thus, when H_2_O (or O_2_) is added to a CH_4_:CO_2_:N_2_ mixture, plasma-based multi-reforming not only allows us to control the chemistry to increase the yield(s) of the desired products(s) (in this case, methanol), but it also allows us to achieve higher conversion rates and thermal efficiencies than standard plasma-based DRM does.

## Enhanced Methanol Yield by Further Tuning Plasma-Based Multi-Reforming

When we added up to 32% H_2_O, the chemistry reached a point of equilibrium, where the methanol selectivity starts to saturate and the initial H_2_O in the mixture begins converting. Therefore, we assumed that adding O_2_ at this point might help to further tune the changes in the plasma chemistry following the tri-reforming principles. So we added several combinations of H_2_O and O_2_. (See the Supplementary Information for complete details). It appeared that the oxidising character of O_2_ remains so dominant that the further enhancement of the plasma chemistry occurs only when a very small amount of O_2_ is added (Table 1).

**Table 1:** Calculated values of effective CH_4_, CO_2_ and H_2_O conversion, methanol (CH_3_OH) selectivity and yield, and thermal efficiency for plasma-based multi-reforming conditions containing varying O_2_ and H_2_O concentrations, for a modelled DBD operating at an SEI of 3 kJ/L, with a mixture of 10% CH_4_ and 10% CO_2_ diluted in N_2_.

Content	Conversion			Selectivity	Yield	Thermal efficiency
H_2_O O_2_	CH_4_ (%)	CO_2_ (%)	H_2_O (%)	CH_3_OH (%)	CH_3_OH (mol%)	η_Thermal, HHV_ (%)
0% H_2_O 0% O_2_	1.01	0.44	n.a.	3.29	0.033	9.43
32% H_2_O 0% O_2_	1.45	0.56	0.21	14.1	0.219	13.85
32% H_2_O 0.5% O_2_	1.76	0.54	0.00	13.7	0.241	14.01
32% H_2_O 1.0% O_2_	2.00	0.52	0.00	12.3	0.246	13.81

When 0.5% O_2_ was added to the mixture containing 32% H_2_O, the effective CH_4_ conversion increased by 21% (from 1.45 to 1.76%), and the effective CO_2_ conversion decreased slightly by 4% (from 0.56 to 0.54%). More importantly, despite a slight decrease in the methanol selectivity from 14.1 to 13.7%, the methanol yield further increased by 10% (from 0.219 to 0.241%). Finally, the thermal efficiency increased by 1.2% (from 13.85 to 14.01%). When we added 1% O_2_, the CH_4_ conversion and methanol yield continued to increase, but the thermal efficiency decreased. Hence, we expect the highest thermal efficiency to occur when O_2_ is added between 0 and 0.5%.

Thus, these results indicate that devising the proper mixture makes it possible to tune the chemical equilibrium of the plasma chemistry in such a way that the reaction pathways, hence the selectivity and yield, towards liquid products can be significantly altered in our favour.

## Role of the Diluting Agent N_2_ and the CH_4_/CO_2_ Ratio

To determine whether the diluting agent N_2_ also affects the chemistry, we changed the N_2_ concentration for a fixed 1:1:3 ratio of CH_4_:CO_2_:H_2_O. For a N_2_ content of 95%, we added 1% CH_4_, 1% CO_2_ and 3% H_2_O; for a N_2_ content of 80%, we added 4% CH_4_, 4% CO_2_ and 12% H_2_O; and for a N_2_ content of 50%, we added 10% CH_4_, 10% CO_2_ and 30% H_2_O. As soon as the N_2_ content was lowered, the thermal efficiency increased drastically, which was not unexpected. Indeed, earlier experiments and model calculations revealed that for high N_2_ contents, a lot of the supplied energy goes into exciting the N_2_ molecules at the expense of dissociating CH_4_ and CO_2_^1,35,36^. Interestingly, however, is the increase in methanol selectivity by more than a factor 2 (from 5.46 to 14.2%), and the increase in methanol yield by more than a factor 6 (from 0.035 to 0.219%), as the N_2_ content decreased from 95 to 50% (Table 2).

**Table 2:** Calculated values of effective CH_4_, CO_2_ and H_2_O conversion, methanol (CH_3_OH) selectivity and yield, and thermal efficiency for plasma-based multi-reforming conditions, varying the concentration of the diluting agent N_2_, for a modelled DBD operating at an SEI of 3 kJ/L, with a fixed 1:1 mixture of CH_4_ and CO_2_.

Content	Conversion			Selectivity	Yield	Thermal efficiency
N_2_ (%)	CH_4_ (%)	CO_2_ (%)	H_2_O (%)	CH_3_OH (%)	CH_3_OH (mol%)	η_Thermal, HHV_ (%)
95	0.62	0.09	0.03	5.46	0.035	5.45
80	0.97	0.27	0.06	10.3	0.103	8.98
50	1.45	0.56	0.18	14.2	0.219	13.73

To explore the effect of the CH_4_/CO_2_ ratio, we varied its value from 0.333 to 3 while keeping the concentration of N_2_ fixed at 80% and that of H_2_O fixed at 10%. We found the highest methanol selectivity for a ratio of 1, while the highest methanol yield was found for a ratio of 3 (Table 3). Additionally, the thermal efficiency increased by 27% (from 7.98 to 10.13%) as the CH_4_/CO_2_ ratio increased from 0.333 to 3. Our investigations lead us to attribute this increase in thermal efficiency to a shift from CO production to C_2_H_6_ production, with the higher methanol yield playing a negligible role.

**Table 3:** Calculated values of effective CH_4_, CO_2_ and H_2_O conversions, methanol (CH_3_OH) selectivity and yield, and thermal efficiency for plasma-based multi-reforming conditions, varying the CH_4_/CO_2_ ratio, for a modelled DBD operating at an SEI of 3 kJ/L, diluted in a fixed N_2_ content of 80%.

Ratio	Conversion			Selectivity	Yield	Thermal efficiency
CH_4_/CO_2_	CH_4_ (%)	CO_2_ (%)	H_2_O (%)	CH_3_OH (%)	CH_3_OH (mol%)	η_Thermal, HHV_ (%)
0.333	0.82	0.48	0.00	8.29	0.068	7.98
1	1.01	0.32	0.01	9.63	0.098	9.26
3	1.12	0.16	0.05	9.19	0.107	10.13

To summarize, in spite of its inert character, the diluting agent N_2_ significantly influences the plasma chemistry. Additionally, the CH_4_/CO_2_ ratio also influences the plasma chemistry and thermal efficiency, but its effect is more nuanced. Depending on the objective a different ratio is needed. (For example to obtain the highest methanol selectivity a different ratio is required than that needed to obtain the highest methanol yield). Thus, these results indicate that many factors can affect the plasma chemistry, and this is why controlling these different parameters is crucial.

## Conclusion and Outlook

We have demonstrated that ‘plasma-based multi-reforming’ is a novel concept that can be successfully applied to improve the gas-to-liquid process. We showed that adding H_2_O and O_2_ to a plasma-based DRM baseline case significantly increases the CH_4_ and CO_2_ conversions, methanol production, and thermal efficiency. These results highlight the benefits of plasma-based multi-reforming over regular plasma-based reforming processes such as DRM and POX.

Our findings suggest that the plasma chemistry can be controlled by adding O_2_ or H_2_O to a CH_4_:CO_2_:N_2_ mixture, and that adding H_2_O yields more promising results. Additionally, we showed that the plasma chemistry can be tuned further if the right combination of initial reactants are added. When we added 32% H_2_O and 0.5% O_2_, the methanol yield increased up to 645%, and the thermal efficiency increased up to 49%, compared to the plasma-based DRM baseline case. Thus, plasma-based multi-reforming appears to have greater significant benefits than those the regular individual reforming processes can offer, similarly to tri-reforming with classic thermocatalysis. Moreover, plasma-based multi-reforming allows to produce methanol through a single-step process; this gives it a huge competitive edge and makes it far more attractive for GTL than the thermocatalytic multi-step process. This shows that in the short term it might be more worthwhile to play with the pure plasma chemistry by working with well thought through additives (O_2_ and H_2_O in this case), without immediately turning to catalyst materials for selectivity enhancements. In the same sense at a later stage the combination of plasma and catalysis will most probably benefit from this approach as well.

These results are promising; but more research is needed. For example, the calculated product yields need further experimental validation and the underlying chemical pathways accessible through the model need to be thoroughly analysed. This data is needed to further unravel the underlying chemical kinetic reaction pathways for us to figure out how we can intervene even more in the chemistry, and eventually to be able to determine the most optimum conditions. Additionally, in this study, we observed that N_2_ significantly alters the chemistry as a diluting agent. Raising the question what is the influence of other diluting agents or mixtures? We also found that the CH_4_/CO_2_ ratio plays a dual role in this story. Which role do we prefer? Temperature dependent studies should be performed as well because product selectivities in plasma-based reforming heavily depend on temperature^26^. Finally, and most importantly, the concept of plasma-based multi-reforming should be explored with more energy efficient and higher throughput reactors, such as gliding arc, microwave and ns-pulsed discharges, as well as their combinations with catalysts^1^. We strongly believe that the effects observed here will also be present in those reactors. Hence, although the current study is limited in scope and we have mountains of work ahead of us, the results presented here are significant in that they give us a sneak peek at the potential of plasma-based multi-reforming and its potentially bright future as GTL technology.

## Electronic supplementary material

Supplementary Information
