# Supplementary material for: Plasma-based multi-reforming for Gas-To-Liquid: tuning the plasma chemistry towards methanol
Source: Sci Rep. 2018 Oct 29;8:15929. doi: 10.1038/s41598-018-34359-x (PMC6206038; doi:10.1038/s41598-018-34359-x)
Supplement: Supplementary file 1 — Supplementary Information [file 41598_2018_34359_MOESM1_ESM.docx]

**Supplementary Information:**

**Plasma-based multi-reforming for Gas-To-Liquid: tuning the plasma chemistry towards methanol**

Ramses Snoeckx,^1,2(a)^ Weizong Wang,^2(a)^ Xuming Zhang,^1(b)^ Min Suk Cha^1^ and Annemie Bogaerts^2^

*^1^King Abdullah University of Science and Technology (KAUST), Clean Combustion Research Center (CCRC), Physical Science and Engineering Division (PSE), Thuwal 23955, Saudi Arabia*

*^2^Research group PLASMANT, Department of Chemistry, University of Antwerp,
Universiteitsplein 1, BE-2610 Antwerp, Belgium*

^(a)^These authors contributed equally

^(b)^New contact affiliation: *College of Environmental Science and Engineering, Zhejiang Gongshang University, Xiasha High Education District, Hangzhou, Zhejiang Province, China*

E-mail: [ramses.snoeckx@kaust.edu.sa](mailto:ramses.snoeckx@kaust.edu.sa) ; [weizong.wang@uantwerpen.be](mailto:weizong.wang@uantwerpen.be)

**1. Description of the experiments**

**1.1. Plasma reactor**

The experimental setup, which was presented in previous studies,^1–3^ consists of a temperature-controlled DBD reactor, a reactant feeding system, a high-voltage power supply, and an analytical system (Figure S1). The temperature-controlled coaxial DBD reactor is made of a quartz tube, an electrical heater furnace, and a high voltage and ground electrode. To configure the coaxial DBD, we install a quartz tube with an inner diameter that contracts from 50 to 20 mm inside the furnace. The wide part of the tube (50 mm) has a length of 450 mm; and is designed to stabilize the temperature of the reactants before they reach the DBD reactor. The length of the contracted part with an inner diameter of 20 mm is 180 mm. The contracted part is used as a dielectric barrier to construct the coaxial DBD reactor. A stainless steel rod with a diameter of 17.5 mm, powered by an adjustable high voltage source of alternating current (AC), is inserted into the contracted section of the quartz tube. A 45 mm wide stainless steel mesh is wrapped around the tube to serve as a ground electrode. This gives a plasma reactor volume of 3.31 cm^3^.


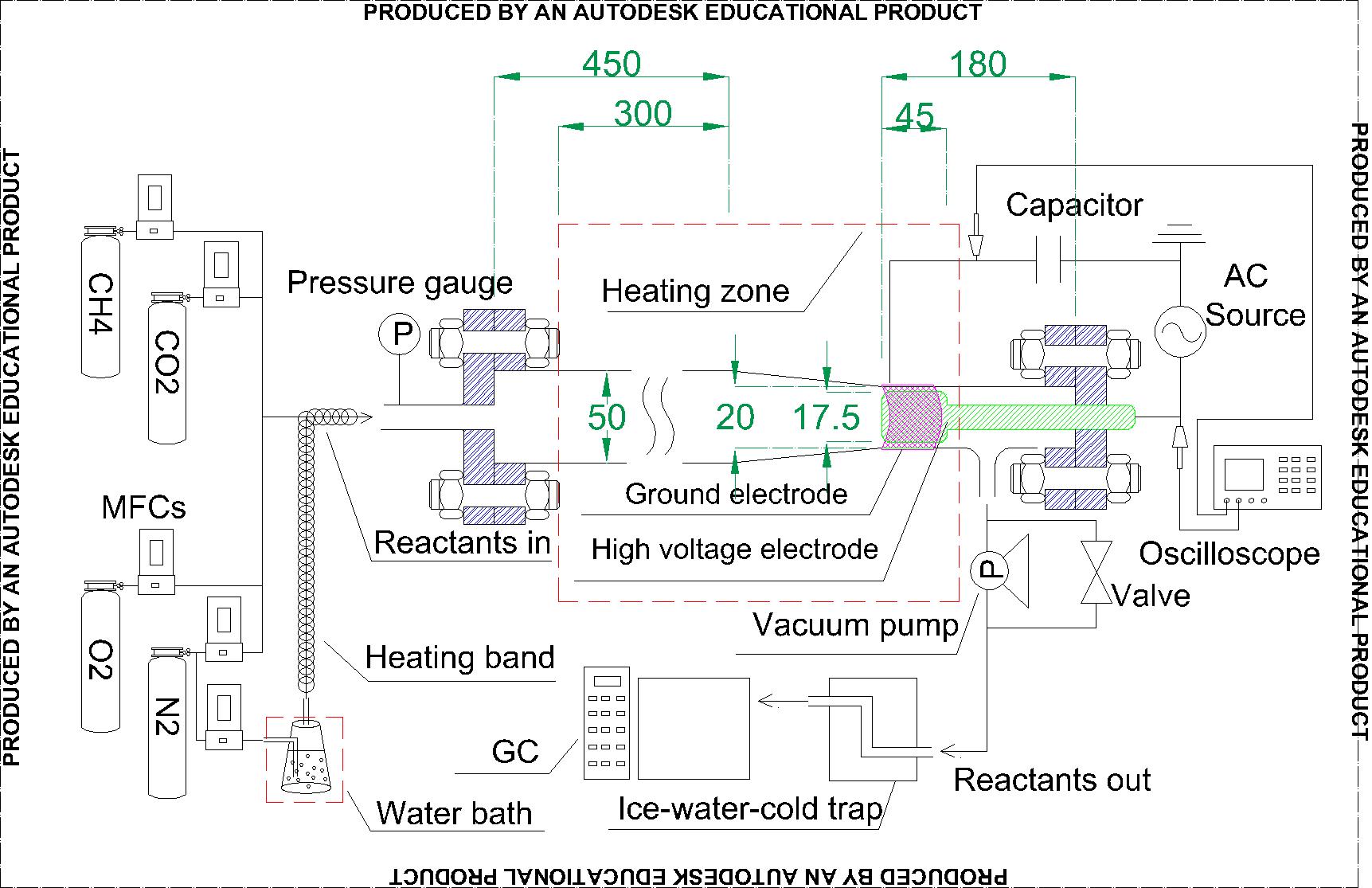


**Figure S1.** Schematic diagram of the experimental setup.

A power amplifier (20/30A, Trek) which can supply voltage and current up to 20 kV and 30 mA, respectively, is used in conjunction with a function generator (AFG 3021B, Tektronix) to generate high voltage AC with a frequency of 3.33 kHz. The applied voltage and discharge current are measured by a digital oscilloscope (DPO 4140B, Tektronix), together with a 1000:1 high voltage probe (P 6015A, Tektronix) and a current monitor (6595, Pearson). We use the *V* –*Q* Lissajous method to determine the discharge power into the DBD reactor. The plasma discharge power is found to be 10 W. The amount of charge, *Q*, is calculated by measuring the voltage across a measuring capacitor (30 nF)—installed in series to the ground line of the DBD reactor—using a 10:1 voltage probe (TPP 1000, Tektronix). The *V* –*Q* Lissajous diagram is averaged over 216 scans, and the discharge power is calculated from the area of the diagram by multiplying the frequency.

The onset voltage (*V*_on_) and the effective capacitances of the barrier (*C*_d_) and the total system (*C*_tot_) can be deduced from the Lissajous diagram (Figure S2). The discharge power can be estimated based on the internal area of the parallelogram and the applied AC frequency, the capacitance of the gas gap (*C*_g_), and the breakdown voltage (*V*_b_) across the gas gap. *E/N* could be determined using the following formula:

$C_{g}=C_{d}C_{tot}/(C_{d}-C_{tot})$ (S1)

$V_{b}=V_{on}/(1+C_{g}/C_{d})$ (S2)

$E/N\approx U_{b}/d/N$ (S3)

**Figure S2.** V-Q Lissajous diagram for an applied peak-to-peak voltage of 20 kV and an AC frequency of 3.3 kHz, at 673 K and 1 bar; resulting power is 10 W.

The total flow rate of the CH_4_:CO_2_:N_2_:O_2_:H_2_O mixtures is fixed at 200 SCCM. The flow rates of methane (CH_4_), carbon dioxide (CO_2_), nitrogen (N_2_) and oxygen (O_2_) are maintained using mass flow controllers (SLA5850, Brooks Instrument), while the amount of water vapour (H_2_O) is controlled by a syringe pump (KDS 100, KD Scientific) and a heater for evaporation. To avoid water condensation, the flow section from the outlet of the heater to the inlet of the temperature controlled DBD reactor is maintained at approximately 400 K. An electric oven was used to heat the gas mixture to 673 K (using thermocouples as feedback), the reactor is operated at atmospheric pressure, and the gas pressure inside the reactor is monitored by a digital pressure gauge (Mano 2000, Keller).

It is important to note that introducing (water) vapour in a DBD always leads to a certain degree of destabilisation of the discharge, making it more difficult to accurately monitor and measure the experimental values. Hence it is very important to have good evaporation and heating of the system. In the current study the discharge did not become too unstable to operate for any condition under study.

**1.2. Product analysis**

To identify the composition of the produced gas, the reformed gas is dried using a homemade ice-water-cooling trap and analysed using an online gas chromatograph (GC; HP 7890A, Agilent) equipped with a flame ionization detector (FID) and two thermal conductivity detectors (TCD).The FID channel is configured to analyse hydrocarbons from C1 to C5. The first TCD channel (reference gas helium) is set to analyse CO_2_, CO and N_2_, and the second TCD channel is used for the detection of H_2_. The gas chromatograph (GC) system is calibrated using reference gas mixtures and He is added as internal standard after the plasma zone to take the gas expansion factor into account as described by Pinhao et al.^4^ In the present study, we do not analyse the condensed liquid fraction, known to contain H_2_O and a variety of alcohols and acids,^5,6^ due to equipment and analysis restrictions. However, we do plan to do this in future studies.

**1.2.1. Conversion**

To characterize the multi-reforming process, the CH_4_, CO_2_, O_2_ and H_2_O conversions (*X*) are defined as:

 (S4)

 (S5)

 (S6)

 (S7)

where represents the molar flow rate of component *i*. Note that the conversion of N_2_ was also monitored with the GC and was found to be negligible as to be expected.

Additionally, we also define the effective conversions (*X_eff_*) of CH_4_, CO_2,_ O_2_ and H_2_O as:

 (S8)

 (S9)

 (S10)

 (S11)

The effective conversion makes it easier to compare the different gas mixtures, as it indicates how much of each component is effectively converted. It also simplifies the formulas used to calculate the product yields (Section 1.2.3).

**1.2.2. Selectivity**

To analyse the products, we define two different selectivities: the H-based selectivity for the H-containing species (i.e. H_2_, H_2_O, CH_3_OH, C_x_H_y_O_z_), and the C-based selectivity for species without (i.e. CO).

For the H-based selectivity:

 (S12)

 (S13)

 (S14)

 (S15)

For mixtures containing H_2_O, the H-based selectivity should be based on both the CH_4_ and H_2_O conversions. However, in practice, for most conditions, more water is generated than converted. As a result, although the initial water is being converted and takes part in the reactions, it does not contribute towards the H-based selectivity, since H_2_O is an H sink instead of a H source in these cases. Therefore, it can be excluded from the H-based selectivity formulas.

For a C-based selectivity:

 (S16) (S17)

In our study, the H-based selectivity can be considered as most important, since we are interested in efficiently converting H from CH_4_ into value-added hydrocarbons; converting it into H_2_O would be an economic and chemical loss.

**1.2.3. Yield**

The yield can be defined based on the effective conversions together with the H-based selectivity or C-based selectivity, or both, depending on the product:

$Y_{\text{H}_{2}}=\left(4\cdot X_{\text{eff,CH}_{4}}+2\cdot X_{\text{eff,H}_{2}\text{O}}\right)\cdot\frac{1}{2}\cdot S_{\text{H,H}_{2}}$ (S18)

$Y_{\text{CH}_{3}\text{OH}}=\left(4\cdot X_{\text{eff,CH}_{4}}+2\cdot X_{\text{eff,H}_{2}\text{O}}\right)\cdot\frac{1}{4}\cdot S_{\text{H,CH}_{3}\text{OH}}=\left(1\cdot X_{\text{eff,CH}_{4}}+1\cdot X_{\text{eff,CO}_{2}}\right)\cdot\frac{1}{1}\cdot S_{\text{C,CH}_{3}\text{OH}}$ (S19)

$Y_{\text{C}_{\text{x}}\text{H}_{\text{y}}\text{O}_{\text{z}}}=\left(4\cdot X_{\text{eff,CH}_{4}}+2\cdot X_{\text{eff,H}_{2}\text{O}}\right)\cdot\frac{1}{y}\cdot S_{\text{H,C}_{\text{x}}\text{H}_{\text{y}}\text{O}_{\text{z}}}=\left(1\cdot X_{\text{eff,CH}_{4}}+1\cdot X_{\text{eff,CO}_{2}}\right)\cdot\frac{1}{x}\cdot S_{\text{C,C}_{\text{x}}\text{H}_{\text{y}}\text{O}_{\text{z}}}$(S20)

$Y_{\text{CO}}=\left(1\cdot X_{\text{eff,CH}_{4}}+1\cdot X_{\text{eff,CO}_{2}}\right)\cdot\frac{1}{1}\cdot S_{\text{C,CO}}$ (S21)

**1.2.4. Thermal efficiency**

To analyse the efficiency of the process, we calculate the thermal efficiency based on the energy output with respect to the energy input:

$\begin{matrix} \eta_{Thermal,HHV}=\frac{E_{out}}{E_{in}} \\ =\frac{\Delta H_{c,fuel}\left(kJ\cdot mol^{-1}\right)}{SEI(kJ\cdot mol^{-1})+\Delta H_{c,convCH_{4}}\left(kJ\cdot mol^{-1}\right)+\left(C_{mol}\left(kJ\cdot mol^{-1}\cdot K^{-1}\right)\cdot T(K)\right)} \end{matrix}$ (S22)

where ΔH_c,fuel_ is the standard enthalpy of combustion of the fuel, based on the high heating value (HHV) of the main products; it represents the energy output. With respect to experimental data, the thermal efficiency is based on the following measured products: H_2_, CO, C_3_H_8_, C_2_H_6_, C_2_H_4_ and C_2_H_2_. For the results from calculations, the thermal efficiency is based on the main products: H_2_, CO, CH_3_OH, C_3_H_8_, C_3_H_6_, C_2_H_6_, C_2_H_4_ and C_2_H_2_ (unless mentioned otherwise in the figure details). The specific energy input (SEI) is based on the plasma power divided by the total gas flow rate (10 W / 200 mL·min^-1^ = 3 kJ L^-1^). We also need to take the HHV of the converted CH_4_ in the feed into account (as part of the denominator), as well as the energy spent for heating the gas to 673 K, since all these elements contribute to the energy input. Table S1 gives an overview of the HHV values used for the different species.

**Table S1.** Overview of the higher heating values (HHV) used for the different species.

| **Species** | **Higher Heating Value (kJ/mol)** |
| --- | --- |
| CO_2_ | 0 |
| CO | 283 |
| CH_4_ | 866 |
| C_3_H_8_ | 2220 |
| C_3_H_6_ | 2057 |
| C_2_H_6_ | 1560 |
| C_2_H_4_ | 1411 |
| C_2_H_2_ | 1310 |
| H_2_ | 286 |
| H_2_O | 0 |
| CH_3_OH | 726 |

**2. Description of the model**

**2.1. 0D chemical kinetics model**

The model used in this work to predict the plasma chemistry is a zero-dimensional (0D) chemical kinetics model, called ZDPlaskin.^7^ In this model, the time evolution of the specie densities is calculated by balance equations, taking into account the various production and loss terms by chemical reactions. Transport processes are not considered; hence, the specie densities are assumed to be constant in the entire simulation volume. Although this means that the plasma is treated as a ‘batch reactor’, we can convert this to represent a ‘plug-flow reactor’ (close to the real situation) by translating the temporal behaviour into a spatial behaviour, as described in section 2.3. below. The rate coefficients of the heavy particle reactions (i.e., atoms, molecules, radicals, ions, and excited species) are assumed to be constant, in accordance to the findings reported in the literature (see section 2.2. below), whereas the rate coefficients for the electron impact reactions are calculated with a Boltzmann solver, BOLSIG+,^8^ which is integrated into ZDPlaskin. A more detailed description of ZDPlaskin is provided by Pancheshnyi et al.^7^

**2.2. Plasma chemistry included in the model**

The chemistry set used in this model was recently developed and validated.^9^ Furthermore, Wang et al. performed an extensive sensitivity analysis to quantify the uncertainties in the modelling results and their impact on the predictive capabilities of the model used in this work.^10^ In short, the model considers 135 different species (see Table S2), which react with each other in 185 electron impact reactions, 651 ion reactions and 809 neutral reactions. The temperature-dependent rate coefficients, electron-impact cross sections and references associated with these data are listed in the Supporting Information of our previous study.^9^

**Table S2.** Overview of the species included in the model.

| **Neutral species** | **Charged species** | **Radical species** | **Excited species** |
| --- | --- | --- | --- |
| CH_4_, C_2_H_2_, C_2_H_4_, C_2_H_6_, C_3_H_6_, C_3_H_8_ | C^+^, CH^+^, CH_2_^+^, CH_3_^+^, CH_4_^+^, CH_5_^+^, C_2_^+^, C_2_H^+^, C_2_H_2_^+^, C_2_H_3_^+^, C_2_H_4_^+^, C_2_H_5_^+^, C_2_H_6_^+^ | C, CH, CH_2_, CH_3_, C_2_, C_2_H_,_ C_2_H_3_, C_2_H_5_, C_3_H_5_, C_3_H_7_, C_4_H_2_ | CH_4_^*^, C_2_H_2_^*^, C_2_H_4_^*^, C_2_H_6_^*^, C_3_H_8_^*^ |
| CO, CO_2_ | CO^+^, CO_2_^+^, CO_4_^+^, C_2_O_2_^+^, C_2_O_3_^+^, C_2_O_4_^+^, CO_3_^-^, CO_4_^-^ | C_2_O |  |
| C_2_N_2_ |  | CN, NCN |  |
| H_2_O, H_2_O_2_ | OH^+^, H_2_O^+^, H_3_O^+^, OH^-^ | OH, HO_2_ | H_2_O^*^ |
| N_2_H_4_, NH_3_, N_2_H_2_ | NH^+^, NH_2_^+^, NH_3_^+^, NH_4_^+^ | NH, NH_2_, N_2_H, N_2_H_3_ | NH_3_^*^ |
| N_2_O, N_2_O_3_, N_2_O_4_, N_2_O_5_ | NO^+^, N_2_O_2_^+^, NO_2_^−^, NO_3_^−^, N_2_O^−^ | NO, NO_2_, NO_3_ |  |
| CH_2_O, CH_3_OH, CH_3_OOH, CH_2_CO, CH_3_CHO, C_2_H_5_OH, C_2_H_5_OOH |  | CHO, CH_2_OH, CH_3_O, CH_3_O_2_, C_2_HO, CH_3_CO, CH_2_CHO, C_2_H_5_O, C_2_H_5_O_2_ |  |
| HCN |  | H_2_CN |  |
| N_2_ | N^+^, N_2_^+^, N_3_^+^, N_4_^+^ | N | N(2P), N(2D), N_2_(V), N_2_(a1), N_2_(C3), N_2_(A3), N_2_(B3) |
| H_2_ | H^+^, H_2_^+^, H_3_^+^, H^−^ | H | H(2P), H_2_(V), H_2_(E), H_2_(V) |
| O_2_, O_3_ | O^+^, O_2_^+^, O_4_^+^, O^−^, O_2_^−^, O_3_^−^, O_4_^−^, | O | O(1D), O(1S), O_2_(a1),O_2_(b1) |

**2.3. Application of the 0D model to a DBD reactor**

As mentioned above, a 0D model calculates densities of species as a function of time only, neglecting spatial variations. However, the time evolution can be translated into a spatial evolution (as a function of position in the DBD reactor) by means of the gas flow rate. This allows us to mimic the typical filamentary behaviour of a DBD used for gas conversion.^5^ Indeed, gas molecules pass through several microdischarge filaments throughout the reactor. This is taken into account in the model by applying a large number of consecutive microdischarge pulses of 30 ns, exactly as described by Bogaerts et al.^11^ This approach has already proven to be applicable for a variety of conditions and gas mixtures.^11–18^

**3. Detailed results**

In this section, we provide detailed input conditions and results for all experiments and calculations.

**3.1. Experimental results**

**3.1.1. Experimental input conditions**

**Table S3.** Overview of the gas mixture compositions used for the different experiments.

|  | **CH_4_** | **CO_2_** | **N_2_** | **H_2_O** | **O_2_** | **CH_4_** | **CO_2_** | **N_2_** | **H_2_O** | **O_2_** |
| --- | --- | --- | --- | --- | --- | --- | --- | --- | --- | --- |
| **No.** | *mL/min* | *mL/min* | *mL/min* | *mL/min* | *mL/min* | *%* | *%* | *%* | *%* | *%* |
| **E1** | 20 | 20 | 160 | 0 | 0 | 10 | 10 | 80 | 0 | 0 |
| *O_2_ addition* | | | | | | *O_2_ addition* | | | | |
| **E2** | 20 | 20 | 156 | 0 | 4 | 10 | 10 | 78 | 0 | 2 |
| **E3** | 20 | 20 | 152 | 0 | 8 | 10 | 10 | 76 | 0 | 4 |
| **E4** | 20 | 20 | 144 | 0 | 16 | 10 | 10 | 72 | 0 | 8 |
| *H_2_O addition* | | | | | | *H_2_O addition* | | | | |
| **E5** | 20 | 20 | 156 | 4 | 0 | 10 | 10 | 78 | 2 | 0 |
| **E6** | 20 | 20 | 152 | 8 | 0 | 10 | 10 | 76 | 4 | 0 |
| **E7** | 20 | 20 | 144 | 16 | 0 | 10 | 10 | 72 | 8 | 0 |

**3.1.2. Experimental results for the addition of O_2_**

**Table S4.** Overview of the experimental CH_4_, CO_2_ and O_2_ conversions as well as the syngas ratio for the experiments with the addition of O_2_.

|  | **Conversion** | | | **Syngas ratio** |
| --- | --- | --- | --- | --- |
|  | **CH_4_** | **CO_2_** | **O_2_** | **H_2_/CO** |
| **No.** | *%* | *%* | *%* |  |
| **E1** | 11.5 | 5.42 | - | 1.30 |
| **E2** | 16.3 | 2.00 | 92.9 | 0.54 |
| **E3** | 18.6 | 1.62 | 63.0 | 0.30 |
| **E4** | 21.4 | 1.95 | 37.7 | 0.20 |

**Table S5.** Overview of the experimental Carbon-based selectivity of the main components and the carbon balance (based on these components) for the experiments with the addition of O_2_.

|  | **Carbon-based Selectivity** | | | | | | **C balance** |
| --- | --- | --- | --- | --- | --- | --- | --- |
|  | **CO** | **C_2_H_6_** | **C_2_H_4_** | **C_2_H_2_** | **C_3_H_8_** | **Other HC’s** |  |
| **No.** | *%* | *%* | *%* | *%* | *%* | *%* | *%* |
| **E1** | 35.4 | 20.0 | 2.00 | 1.50 | 5.50 | 1.30 | 65.7 |
| **E2** | 78.5 | 8.27 | 0.77 | 0.53 | 0.52 | 0.00 | 88.6 |
| **E3** | 97.0 | 3.05 | 0.67 | 0.46 | 0.00 | 0.00 | 101.2 |
| **E4** | 98.8 | 1.24 | 0.46 | 0.25 | 0.00 | 0.00 | 100.8 |

**Table S6.** Overview of the experimental Hydrogen-based selectivity of the main components and the hydrogen balance (based on these components) for the experiments with the addition of O_2_.

|  | **Hydrogen-based Selectivity** | | | | | **H balance** |
| --- | --- | --- | --- | --- | --- | --- |
|  | **H_2_** | **C_2_H_6_** | **C_2_H_4_** | **C_2_H_2_** | **C_3_H_8_** |  |
| **No.** | *%* | *%* | *%* | *%* | *%* | *%* |
| **E1** | 32.0 | 22.1 | 1.47 | 0.55 | 5.40 | 61.5 |
| **E2** | 22.2 | 6.96 | 0.43 | 0.15 | 0.39 | 30.2 |
| **E3** | 13.8 | 2.49 | 0.36 | 0.13 | 0.00 | 16.8 |
| **E4** | 9.28 | 1.01 | 0.25 | 0.07 | 0.00 | 10.6 |

**3.1.3. Experimental results for the addition of H_2_O**

**Table S7.** Overview of the experimental CH_4_, CO_2_ and H_2_O conversions as well as the syngas ratio for the experiments with the addition of H_2_O.

|  | **Conversion** | | | **Syngas ratio** |
| --- | --- | --- | --- | --- |
|  | **CH_4_** | **CO_2_** | **H_2_O** | **H_2_/CO** |
| **No.** | *%* | *%* | *%* |  |
| **E1** | 11.5 | 5.42 | - | 1.30 |
| **E5** | 12.7 | 4.56 | n.a. | 1.43 |
| **E6** | 12.1 | 4.49 | n.a. | 1.53 |
| **E7** | 11.4 | 5.05 | n.a. | 1.68 |

**Table S8.** Overview of the experimental Carbon-based selectivity of the main components and the carbon balance for the experiments with the addition of H_2_O.

|  | **Carbon-based Selectivity** | | | | | | **C balance** |
| --- | --- | --- | --- | --- | --- | --- | --- |
|  | **CO** | **C_2_H_6_** | **C_2_H_4_** | **C_2_H_2_** | **C_3_H_8_** | **Other HC’s** |  |
| **No.** | *%* | *%* | *%* | *%* | *%* | *%* | *%* |
| **E1** | 35.4 | 20.0 | 2.00 | 1.50 | 5.50 | 1.30 | 65.7 |
| **E5** | 37.7 | 21.6 | 1.70 | 1.30 | 5.40 | 1.10 | 68.8 |
| **E6** | 38.6 | 19.9 | 1.30 | 0.95 | 5.00 | 1.30 | 67.1 |
| **E7** | 40.4 | 22.4 | 1.30 | 1.00 | 5.90 | 1.20 | 72.2 |

**Table S9.** Overview of the experimental Hydrogen-based selectivity of the main components and the carbon balance for the experiments with the addition of H_2_O.

|  | **Hydrogen-based Selectivity** | | | | | **H balance** |
| --- | --- | --- | --- | --- | --- | --- |
|  | **H_2_** | **C_2_H_6_** | **C_2_H_4_** | **C_2_H_2_** | **C_3_H_8_** |  |
| **No.** | *%* | *%* | *%* | *%* | *%* | *%* |
| **E1** | 32.0 | 22.1 | 1.47 | 0.55 | 5.40 | 61.5 |
| **E5** | 39.8 | 22.0 | 1.16 | 0.44 | 4.89 | 68.3 |
| **E6** | 42.8 | 20.7 | 0.90 | 0.33 | 4.63 | 69.4 |
| **E7** | 47.3 | 24.3 | 0.94 | 0.36 | 5.68 | 78.6 |

**3.2. Modelling results**

**3.2.1. Modelling input conditions**

**Table S10.** Overview of the gas mixture compositions used for the different modelling cases.

|  | **CH_4_** | **CO_2_** | **N_2_** | **H_2_O** | **O_2_** | **CH_4_** | **CO_2_** | **N_2_** | **H_2_O** | **O_2_** |
| --- | --- | --- | --- | --- | --- | --- | --- | --- | --- | --- |
| **No.** | *mL/min* | *mL/min* | *mL/min* | *mL/min* | *mL/min* | *%* | *%* | *%* | *%* | *%* |
| **M1** | 20 | 20 | 160 | 0 | 0 | 10 | 10 | 80 | 0 | 0 |
| *O_2_ addition* | | | | | | *O_2_ addition* | | | | |
| **M2** | 20 | 20 | 156 | 0 | 4 | 10 | 10 | 78 | 0 | 2 |
| **M3** | 20 | 20 | 152 | 0 | 8 | 10 | 10 | 76 | 0 | 4 |
| **M4** | 20 | 20 | 144 | 0 | 16 | 10 | 10 | 72 | 0 | 8 |
| **M5** | 20 | 20 | 128 | 0 | 32 | 10 | 10 | 64 | 0 | 16 |
| **M6** | 20 | 20 | 96 | 0 | 64 | 10 | 10 | 48 | 0 | 32 |
| *H_2_O addition* | | | | | | *H_2_O addition* | | | | |
| **M7** | 20 | 20 | 156 | 4 | 0 | 10 | 10 | 78 | 2 | 0 |
| **M8** | 20 | 20 | 152 | 8 | 0 | 10 | 10 | 76 | 4 | 0 |
| **M9** | 20 | 20 | 144 | 16 | 0 | 10 | 10 | 72 | 8 | 0 |
| **M10** | 20 | 20 | 128 | 32 | 0 | 10 | 10 | 64 | 16 | 0 |
| **M11** | 20 | 20 | 96 | 64 | 0 | 10 | 10 | 48 | 32 | 0 |
| *Combined H_2_O and O_2_ addition* | | | | | | *Combined H_2_O and O_2_ addition* | | | | |
| **M12** | 20 | 20 | 152 | 4 | 4 | 10 | 10 | 76 | 2 | 2 |
| **M13** | 20 | 20 | 148 | 6 | 6 | 10 | 10 | 74 | 3 | 3 |
| **M14** | 20 | 20 | 148 | 4 | 8 | 10 | 10 | 74 | 2 | 4 |
| **M15** | 20 | 20 | 148 | 8 | 4 | 10 | 10 | 74 | 4 | 2 |
|  |  |  |  |  |  |  |  |  |  |  |
| **M16** | 20 | 20 | 150 | 8 | 2 | 10 | 10 | 75 | 4 | 1 |
| **M17** | 20 | 20 | 142 | 16 | 2 | 10 | 10 | 71 | 8 | 1 |
| **M18** | 20 | 20 | 126 | 32 | 2 | 10 | 10 | 63 | 16 | 1 |
| **M19** | 20 | 20 | 94 | 64 | 2 | 10 | 10 | 47 | 32 | 1 |
|  |  |  |  |  |  |  |  |  |  |  |
| **M20** | 20 | 20 | 94.5 | 64 | 1 | 10 | 10 | 47.25 | 32 | 0.75 |
| **M21** | 20 | 20 | 95 | 64 | 1.5 | 10 | 10 | 47.5 | 32 | 0.5 |
| *Varying N_2_* | | | | | | *Varying N_2_* | | | | |
| **M22** | 2 | 2 | 190 | 6 | 0 | 1 | 1 | 95 | 3 | 0 |
| **M23** | 8 | 8 | 160 | 24 | 0 | 4 | 4 | 80 | 12 | 0 |
| **M24** | 20 | 20 | 100 | 60 | 0 | 10 | 10 | 50 | 30 | 0 |
| *Varying CH_4_/CO_2_ ratio* | | | | | | *Varying CH_4_/CO_2_ ratio* | | | | |
| **M25** | 5 | 15 | 160 | 20 | 0 | 2.5 | 7.5 | 80 | 10 | 0 |
| **M26** | 10 | 10 | 160 | 20 | 0 | 5 | 5 | 80 | 10 | 0 |
| **M27** | 15 | 5 | 160 | 20 | 0 | 7.5 | 2.5 | 80 | 10 | 0 |

**3.2.2. Modelling results for the addition of O_2_**

**Table S11.** Overview of the calculated CH_4_, CO_2_ and O_2_ conversions as well as the syngas ratio for the modelling cases with the addition of O_2_.

|  | **Conversion** | | | **Syngas ratio** |
| --- | --- | --- | --- | --- |
|  | **CH_4_** | **CO_2_** | **O_2_** | **H_2_/CO** |
| **No.** | *%* | *%* | *%* |  |
| **M1** | 10.1 | 4.38 | - | 1.34 |
| **M2** | 16.7 | 3.53 | 62.3 | 0.59 |
| **M3** | 20.8 | 3.18 | 50.7 | 0.39 |
| **M4** | 25.4 | 2.65 | 36.7 | 0.25 |
| **M5** | 30.2 | 1.95 | 24.5 | 0.17 |
| **M6** | 36.4 | 1.13 | 16.0 | 0.11 |

**Table S12.** Overview of the calculated Carbon-based selectivity of the main components and the carbon balance (based on these components) for the modelling cases with the addition of O_2_.

|  | **Carbon-based Selectivity** | | | | | | | | **C balance** |
| --- | --- | --- | --- | --- | --- | --- | --- | --- | --- |
|  | **CO** | **CH_3_OH** | **CH_3_CHO** | **C_2_H_6_** | **C_2_H_4_** | **C_2_H_2_** | **C_3_H_8_** | **HCN** |  |
| **No.** | *%* | *%* | *%* | *%* | *%* | *%* | *%* | *%* | *%* |
| **M1** | 41.8 | 2.29 | 3.83 | 28.1 | 1.91 | 1.57 | 5.76 | 11.7 | 96.9 |
| **M2** | 65.3 | 6.32 | 0.83 | 12.8 | 0.41 | 0.27 | 0.98 | 4.67 | 91.5 |
| **M3** | 73.0 | 6.44 | 0.39 | 8.29 | 0.30 | 0.15 | 0.41 | 2.42 | 91.4 |
| **M4** | 80.5 | 5.88 | 0.17 | 4.65 | 0.23 | 0.08 | 0.13 | 1.07 | 93.4 |
| **M5** | 86.8 | 4.70 | 0.06 | 2.31 | 0.17 | 0.03 | 0.03 | 0.48 | 94.6 |
| **M6** | 92.1 | 3.16 | 0.02 | 0.92 | 0.10 | 0.01 | 0.01 | 0.22 | 96.5 |

**Table S13.** Overview of the calculated Hydrogen-based selectivity of the main components and the hydrogen balance (based on these components) for the modelling cases with the addition of O_2_.

|  | **Hydrogen-based Selectivity** | | | | | | | | | **H balance** |
| --- | --- | --- | --- | --- | --- | --- | --- | --- | --- | --- |
|  | **H_2_** | **H_2_O** | **CH_3_OH** | **CH_3_CHO** | **C_2_H_6_** | **C_2_H_4_** | **C_2_H_2_** | **C_3_H_8_** | **HCN** |  |
| **No.** | *%* | *%* | *%* | *%* | *%* | *%* | *%* | *%* | *%* | *%* |
| **M1** | 40.2 | 9.65 | 3.29 | 2.75 | 30.2 | 1.37 | 0.56 | 5.51 | 4.20 | 97.8 |
| **M2** | 23.4 | 47.4 | 7.65 | 0.50 | 11.6 | 0.25 | 0.08 | 0.79 | 1.41 | 93.1 |
| **M3** | 16.4 | 60.4 | 7.43 | 0.23 | 7.2 | 0.17 | 0.04 | 0.32 | 0.70 | 92.8 |
| **M4** | 11.2 | 71.3 | 6.49 | 0.17 | 3.9 | 0.13 | 0.02 | 0.10 | 0.30 | 92.7 |
| **M5** | 7.79 | 79.3 | 5.01 | 0.03 | 1.8 | 0.09 | 0.01 | 0.02 | 0.13 | 94.3 |
| **M6** | 5.36 | 85.6 | 3.26 | 0.01 | 0.71 | 0.05 | 0.00 | 0.00 | 0.06 | 95.0 |

**3.2.3. Modelling results for the addition of H_2_O**

**Table S14.** Overview of the calculated CH_4_, CO_2_ and H_2_O conversions as well as the syngas ratio for the modelling cases with the addition of H_2_O.

|  | **Conversion** | | | **Syngas ratio** |
| --- | --- | --- | --- | --- |
|  | **CH_4_** | **CO_2_** | **H_2_O** | **H_2_/CO** |
| **No.** | *%* | *%* | *%* |  |
| **M1** | 10.1 | 4.38 | - | 1.34 |
| **M7** | 10.6 | 4.57 | n.a. | 1.38 |
| **M8** | 11.1 | 4.71 | n.a. | 1.42 |
| **M9** | 12.0 | 4.97 | n.a. | 1.50 |
| **M10** | 13.4 | 5.35 | n.a. | 1.62 |
| **M11** | 14.5 | 5.64 | 0.65 | 1.76 |

**Table S15.** Overview of the calculated Carbon-based selectivity of the main components and the carbon balance (based on these components) for the modelling cases with the addition of H_2_O.

|  | **Carbon-based Selectivity** | | | | | | | | **C balance** |
| --- | --- | --- | --- | --- | --- | --- | --- | --- | --- |
|  | **CO** | **CH_3_OH** | **CH_3_CHO** | **C_2_H_6_** | **C_2_H_4_** | **C_2_H_2_** | **C_3_H_8_** | **HCN** |  |
| **No.** | *%* | *%* | *%* | *%* | *%* | *%* | *%* | *%* | *%* |
| **M1** | 41.8 | 2.29 | 3.83 | 28.1 | 1.91 | 1.57 | 5.76 | 11.7 | 96.9 |
| **M7** | 42.0 | 3.39 | 3.65 | 27.2 | 1.78 | 1.48 | 5.48 | 11.9 | 96.8 |
| **M8** | 42.1 | 4.33 | 3.47 | 26.4 | 1.67 | 1.41 | 5.26 | 12.1 | 96.8 |
| **M9** | 42.6 | 6.09 | 3.10 | 25.1 | 1.43 | 1.24 | 4.78 | 12.4 | 96.7 |
| **M10** | 44.4 | 8.81 | 2.35 | 22.8 | 0.93 | 0.91 | 3.79 | 12.8 | 96.8 |
| **M11** | 51.3 | 10.85 | 1.27 | 18.8 | 0.24 | 0.38 | 2.10 | 12.1 | 97.1 |

**Table S16.** Overview of the calculated Hydrogen-based selectivity of the main components and the hydrogen balance (based on these components) for the modelling cases with the addition of H_2_O.

|  | **Hydrogen-based Selectivity** | | | | | | | | | **H balance** |
| --- | --- | --- | --- | --- | --- | --- | --- | --- | --- | --- |
|  | **H_2_** | **H_2_O** | **CH_3_OH** | **CH_3_CHO** | **C_2_H_6_** | **C_2_H_4_** | **C_2_H_2_** | **C_3_H_8_** | **HCN** |  |
| **No.** | *%* | *%* | *%* | *%* | *%* | *%* | *%* | *%* | *%* | *%* |
| **M1** | 40.2 | 9.65 | 3.29 | 2.75 | 30.2 | 1.37 | 0.56 | 5.51 | 4.20 | 97.8 |
| **M7** | 41.5 | 8.31 | 4.84 | 2.61 | 29.1 | 1.28 | 0.53 | 5.23 | 4.27 | 97.7 |
| **M8** | 42.7 | 7.09 | 6.18 | 2.48 | 28.3 | 1.19 | 0.50 | 4.99 | 4.31 | 97.7 |
| **M9** | 45.2 | 4.72 | 8.62 | 2.20 | 26.6 | 1.01 | 0.44 | 4.51 | 4.39 | 97.7 |
| **M10** | 50.4 | 0.38 | 12.34 | 1.65 | 23.9 | 0.65 | 0.32 | 3.53 | 4.48 | 97.7 |
| **M11** | 58.6 | n.a. | 14.07 | 0.82 | 18.3 | 0.16 | 0.12 | 1.81 | 3.93 | 97.7 |

**3.2.4. Modelling results for the combined addition of O_2_ and H_2_O**

**Table S17.** Overview of the calculated CH_4_, CO_2_, O_2_ and H_2_O conversions as well as the syngas ratio for the modelling cases with the combined addition of O_2_ and H_2_O.

|  | **Conversion** | | | | **Syngas ratio** |
| --- | --- | --- | --- | --- | --- |
|  | **CH_4_** | **CO_2_** | **O_2_** | **H_2_O** | **H_2_/CO** |
| **No.** | *%* | *%* | *%* | *%* |  |
| **M1** | 10.1 | 4.38 | - | - | 1.34 |
| **M12** | 17.5 | 3.67 | 66.0 | n.a. | 0.60 |
| **M13** | 20.3 | 3.52 | 60.2 | n.a. | 0.47 |
| **M14** | 21.7 | 3.29 | 53.2 | n.a. | 0.39 |
| **M15** | 18.2 | 3.80 | 68.8 | n.a. | 0.61 |
|  |  |  |  |  |  |
| **M16** | 15.0 | 4.10 | 78.1 | n.a. | 0.86 |
| **M17** | 16.1 | 4.37 | 82.6 | n.a. | 0.90 |
| **M18** | 18.0 | 4.80 | 88.9 | n.a. | 0.98 |
| **M19** | 20.0 | 5.18 | 94.9 | n.a. | 1.10 |
|  |  |  |  |  |  |
| **M20** | 17.6 | 5.43 | 95.7 | n.a. | 1.38 |
| **M21** | 18.8 | 5.31 | 95.5 | n.a. | 1.23 |

**Table S18.** Overview of the calculated Carbon-based selectivity of the main components and the carbon balance (based on these components) for the modelling cases with the addition of O_2_ and H_2_O.

|  | **Carbon-based Selectivity** | | | | | | | | **C balance** |
| --- | --- | --- | --- | --- | --- | --- | --- | --- | --- |
|  | **CO** | **CH_3_OH** | **CH_3_CHO** | **C_2_H_6_** | **C_2_H_4_** | **C_2_H_2_** | **C_3_H_8_** | **HCN** |  |
| **No.** | *%* | *%* | *%* | *%* | *%* | *%* | *%* | *%* | *%* |
| **M1** | 41.8 | 2.29 | 3.83 | 28.1 | 1.91 | 1.57 | 5.76 | 11.7 | 96.9 |
| **M12** | 65.7 | 6.50 | 0.82 | 12.2 | 0.39 | 0.28 | 0.99 | 4.88 | 91.7 |
| **M13** | 70.4 | 6.59 | 0.54 | 9.34 | 0.32 | 0.19 | 0.62 | 3.49 | 91.5 |
| **M14** | 73.5 | 6.46 | 0.39 | 7.83 | 0.29 | 0.15 | 0.41 | 2.53 | 91.5 |
| **M15** | 65.9 | 6.68 | 0.82 | 11.6 | 0.38 | 0.28 | 1.00 | 5.08 | 91.8 |
|  |  |  |  |  |  |  |  |  |  |
| **M16** | 58.8 | 6.39 | 1.45 | 15.9 | 0.59 | 0.49 | 1.85 | 7.69 | 93.1 |
| **M17** | 58.8 | 7.15 | 1.41 | 15.0 | 0.56 | 0.49 | 1.87 | 8.13 | 93.4 |
| **M18** | 59.0 | 8.44 | 1.24 | 13.7 | 0.44 | 0.46 | 1.82 | 8.83 | 94.0 |
| **M19** | 61.5 | 9.77 | 0.81 | 12.1 | 0.18 | 0.29 | 1.42 | 9.06 | 96.0 |
|  |  |  |  |  |  |  |  |  |  |
| **M20** | 56.7 | 10.5 | 0.99 | 15.0 | 0.21 | 0.34 | 1.75 | 10.7 | 96.1 |
| **M21** | 59.1 | 10.1 | 0.89 | 13.5 | 0.20 | 0.31 | 1.58 | 9.88 | 95.6 |

**Table S19.** Overview of the calculated Hydrogen-based selectivity of the main components and the hydrogen balance (based on these components) for the modelling cases with the addition of O_2_ and H_2_O.

|  | **Hydrogen-based Selectivity** | | | | | | | | | **H balance** |
| --- | --- | --- | --- | --- | --- | --- | --- | --- | --- | --- |
|  | **H_2_** | **H_2_O** | **CH_3_OH** | **CH_3_CHO** | **C_2_H_6_** | **C_2_H_4_** | **C_2_H_2_** | **C_3_H_8_** | **HCN** |  |
| **No.** | *%* | *%* | *%* | *%* | *%* | *%* | *%* | *%* | *%* | *%* |
| **M1** | 40.2 | 9.65 | 3.29 | 2.75 | 30.2 | 1.37 | 0.56 | 5.51 | 4.20 | 97.8 |
| **M12** | 24.0 | 47.3 | 7.87 | 0.50 | 11.1 | 0.24 | 0.08 | 0.80 | 1.48 | 93.2 |
| **M13** | 19.6 | 55.3 | 7.73 | 0.31 | 8.22 | 0.19 | 0.06 | 0.48 | 1.03 | 92.9 |
| **M14** | 16.6 | 60.6 | 7.44 | 0.22 | 6.76 | 0.17 | 0.04 | 0.32 | 0.73 | 92.9 |
| **M15** | 24.4 | 47.1 | 8.07 | 0.49 | 10.6 | 0.23 | 0.08 | 0.81 | 1.54 | 93.3 |
|  |  |  |  |  |  |  |  |  |  |  |
| **M16** | 32.2 | 33.5 | 8.14 | 0.92 | 15.2 | 0.38 | 0.16 | 1.57 | 2.45 | 94.5 |
| **M17** | 33.7 | 32.1 | 9.10 | 0.89 | 14.3 | 0.35 | 0.16 | 1.59 | 2.59 | 94.7 |
| **M18** | 36.6 | 29.3 | 10.7 | 0.78 | 13.1 | 0.28 | 0.14 | 1.54 | 2.80 | 95.2 |
| **M19** | 42.5 | 25.0 | 12.3 | 0.51 | 11.5 | 0.11 | 0.09 | 1.19 | 2.85 | 95.2 |
|  |  |  |  |  |  |  |  |  |  |  |
| **M20** | 51.1 | 11.4 | 13.7 | 0.65 | 14.7 | 0.14 | 0.11 | 1.53 | 3.52 | 96.9 |
| **M21** | 46.5 | 18.7 | 13.0 | 0.57 | 12.9 | 0.13 | 0.10 | 1.35 | 3.17 | 96.4 |

**3.2.5. Modelling results obtained by varying N_2_ content**

**Table S20.** Overview of the calculated CH_4_, CO_2_, H_2_O conversions as well as the syngas ratio for the modelling cases with the addition of H_2_O for varying N_2_ content.

|  | **Conversion** | | | **Syngas ratio** |
| --- | --- | --- | --- | --- |
|  | **CH_4_** | **CO_2_** | **H_2_O** | **H_2_/CO** |
| **No.** | *%* | *%* | *%* |  |
| **M1** | 10.1 | 4.38 | - | 1.34 |
| **M22** | 62.4 | 8.79 | 0.85 | 5.03 |
| **M23** | 24.3 | 6.82 | 0.48 | 2.38 |
| **M24** | 14.5 | 5.63 | 0.60 | 1.75 |

**Table S21.** Overview of the calculated Carbon-based selectivity of the main components and the carbon balance (based on these components) for the modelling cases with the addition of H_2_O for varying N_2_ content.

|  | **Carbon-based Selectivity** | | | | | | | | **C balance** |
| --- | --- | --- | --- | --- | --- | --- | --- | --- | --- |
|  | **CO** | **CH_3_OH** | **CH_3_CHO** | **C_2_H_6_** | **C_2_H_4_** | **C_2_H_2_** | **C_3_H_8_** | **HCN** |  |
| **No.** | *%* | *%* | *%* | *%* | *%* | *%* | *%* | *%* | *%* |
| **M1** | 41.8 | 2.29 | 3.83 | 28.1 | 1.91 | 1.57 | 5.76 | 11.7 | 96.9 |
| **M22** | 20.2 | 4.89 | 3.31 | 14.2 | 4.27 | 5.27 | 11.2 | 27.4 | 90.6 |
| **M23** | 36.5 | 8.30 | 2.58 | 21.3 | 1.54 | 1.73 | 5.04 | 19.1 | 96.1 |
| **M24** | 50.3 | 10.8 | 1.37 | 19.3 | 0.30 | 0.43 | 2.28 | 12.33 | 97.1 |

**Table S22.** Overview of the calculated Hydrogen-based selectivity of the main components and the hydrogen balance (based on these components) for the modelling cases with the addition of H_2_O for varying N_2_ content.

|  | **Hydrogen-based Selectivity** | | | | | | | | | **H balance** |
| --- | --- | --- | --- | --- | --- | --- | --- | --- | --- | --- |
|  | **H_2_** | **H_2_O** | **CH_3_OH** | **CH_3_CHO** | **C_2_H_6_** | **C_2_H_4_** | **C_2_H_2_** | **C_3_H_8_** | **HCN** |  |
| **No.** | *%* | *%* | *%* | *%* | *%* | *%* | *%* | *%* | *%* | *%* |
| **M1** | 40.2 | 9.65 | 3.29 | 2.75 | 30.2 | 1.37 | 0.56 | 5.51 | 4.20 | 97.8 |
| **M22** | 56.7 | n.a. | 5.46 | 1.85 | 11.9 | 2.39 | 1.47 | 8.33 | 7.65 | 95.7 |
| **M23** | 54.1 | n.a. | 10.3 | 1.60 | 19.9 | 0.95 | 0.54 | 4.18 | 5.92 | 97.5 |
| **M24** | 57.5 | n.a. | 14.2 | 0.89 | 18.9 | 0.20 | 0.14 | 1.98 | 4.03 | 97.8 |

**3.2.6. Modelling results obtained by varying the CH_4_/CO_2_ ratio**

**Table S23.** Overview of the calculated CH_4_, CO_2_, H_2_O conversions as well as the syngas ratio for the modelling cases with the addition of H_2_O for varying CH_4_/CO_2_ ratio.

|  | **Conversion** | | | **Syngas ratio** |
| --- | --- | --- | --- | --- |
|  | **CH_4_** | **CO_2_** | **H_2_O** | **H_2_/CO** |
| **No.** | *%* | *%* | *%* |  |
| **M1** | 10.1 | 4.38 | - | 1.34 |
| **M25** | 32.8 | 6.40 | n.a. | 1.28 |
| **M26** | 20.1 | 6.30 | 0.11 | 2.13 |
| **M27** | 14.9 | 6.25 | 0.84 | 4.25 |

**Table S24.** Overview of the calculated Carbon-based selectivity of the main components and the carbon balance (based on these components) for the modelling cases with the addition of H_2_O for varying CH_4_/CO_2_ ratio.

|  | **Carbon-based Selectivity** | | | | | | | | **C balance** |
| --- | --- | --- | --- | --- | --- | --- | --- | --- | --- |
|  | **CO** | **CH_3_OH** | **CH_3_CHO** | **C_2_H_6_** | **C_2_H_4_** | **C_2_H_2_** | **C_3_H_8_** | **HCN** |  |
| **No.** | *%* | *%* | *%* | *%* | *%* | *%* | *%* | *%* | *%* |
| **M1** | 41.8 | 2.29 | 3.83 | 28.1 | 1.91 | 1.57 | 5.76 | 11.7 | 96.9 |
| **M25** | 58.9 | 5.23 | 2.46 | 11.7 | 0.63 | 0.77 | 2.46 | 15.1 | 97.3 |
| **M26** | 37.6 | 7.37 | 2.90 | 22.4 | 1.60 | 1.69 | 5.07 | 17.6 | 96.3 |
| **M27** | 20.1 | 8.36 | 2.28 | 32.6 | 2.71 | 2.68 | 7.95 | 18.6 | 95.3 |

**Table S25.** Overview of the calculated Hydrogen-based selectivity of the main components and the hydrogen balance (based on these components) for the modelling cases with the addition of H_2_O for varying CH_4_/CO_2_ ratio.

|  | **Hydrogen-based Selectivity** | | | | | | | | | **H balance** |
| --- | --- | --- | --- | --- | --- | --- | --- | --- | --- | --- |
|  | **H_2_** | **H_2_O** | **CH_3_OH** | **CH_3_CHO** | **C_2_H_6_** | **C_2_H_4_** | **C_2_H_2_** | **C_3_H_8_** | **HCN** |  |
| **No.** | *%* | *%* | *%* | *%* | *%* | *%* | *%* | *%* | *%* | *%* |
| **M1** | 40.2 | 9.65 | 3.29 | 2.75 | 30.2 | 1.37 | 0.56 | 5.51 | 4.20 | 97.8 |
| **M25** | 59.7 | 4.35 | 8.29 | 1.95 | 13.9 | 0.50 | 0.31 | 2.60 | 6.00 | 97.6 |
| **M26** | 52.3 | n.a. | 9.63 | 2.90 | 21.9 | 1.04 | 0.55 | 4.41 | 5.74 | 97.5 |
| **M27** | 47 | n.a. | 9.19 | 1.25 | 26.8 | 1.49 | 0.74 | 5.82 | 5.10 | 97.4 |

**3.3. Calculated yields for the additions of O_2_ and H_2_O**

This section presents the calculated yields for the additions of O_2_ and H_2_O, based on the modelled conversions and selectivities provided in the main manuscript (see also tables above). The trends observed for the yield are similar to those observed for the selectivity (and discussed in the main manuscript), as the yield is the product of the effective conversion and the selectivity.

Upon addition of O_2_, the CO and H_2_O yields both increase dramatically, mainly at the expense of the H_2_ and C_2_H_6_ yields. At first, the CH_3_OH yield increases drastically upon addition of 2 % of O_2_, but this increase rapidly tops off at 8 % O_2_ and starts decreasing as well from that point on.

Upon addition of H_2_O, the C_2_H_6_ yield remains relatively constant, with a slight decrease when adding H_2_O up to 32 %. The H_2_O yield decreases rapidly and, for the range 16–32 % of added H_2_O, there is no more net formation of H_2_O. H_2_ and CO yields increase linearly, while the CH_3_OH increases rapidly, but starts deviating from its linear increase upon addition of 32 % H_2_O.


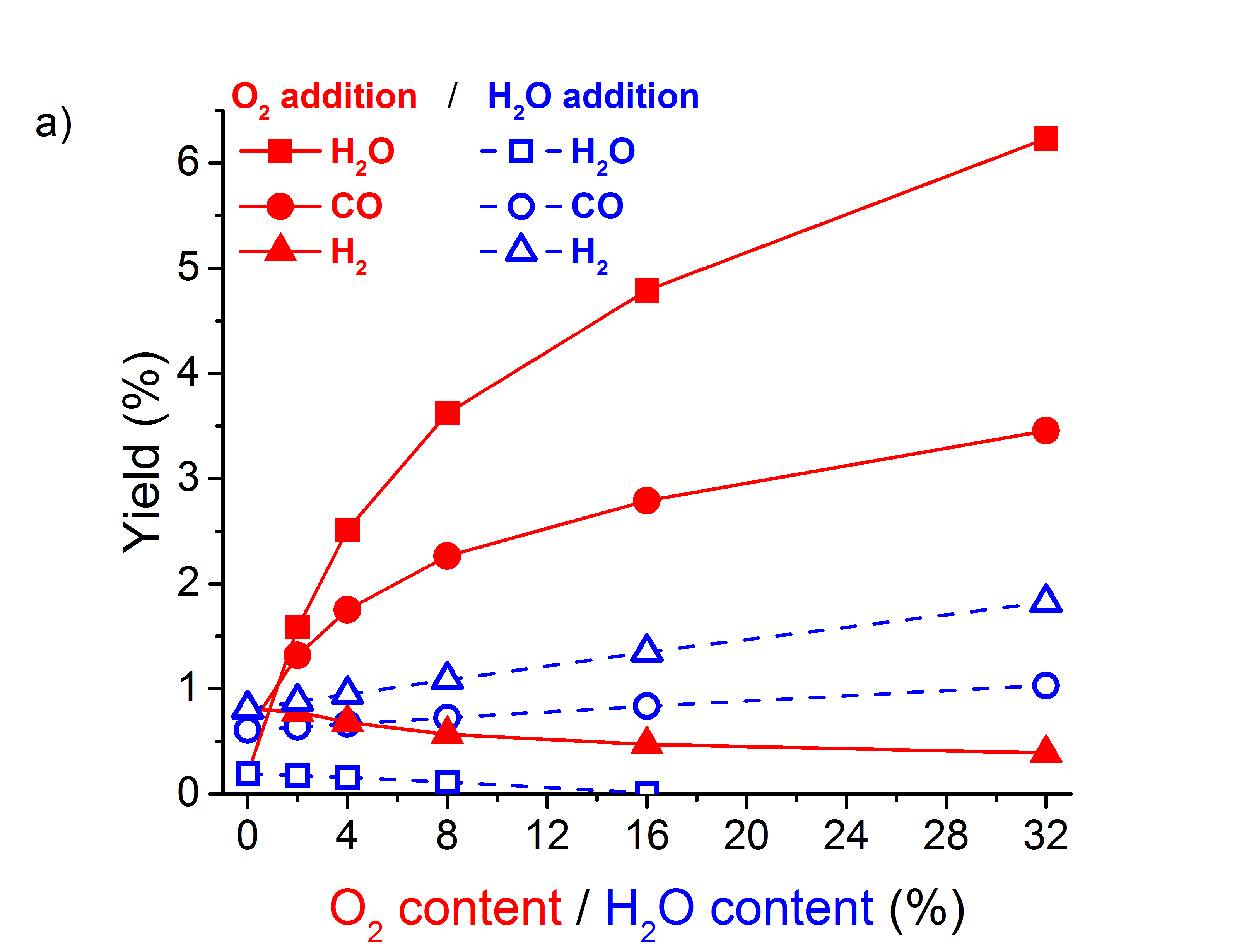

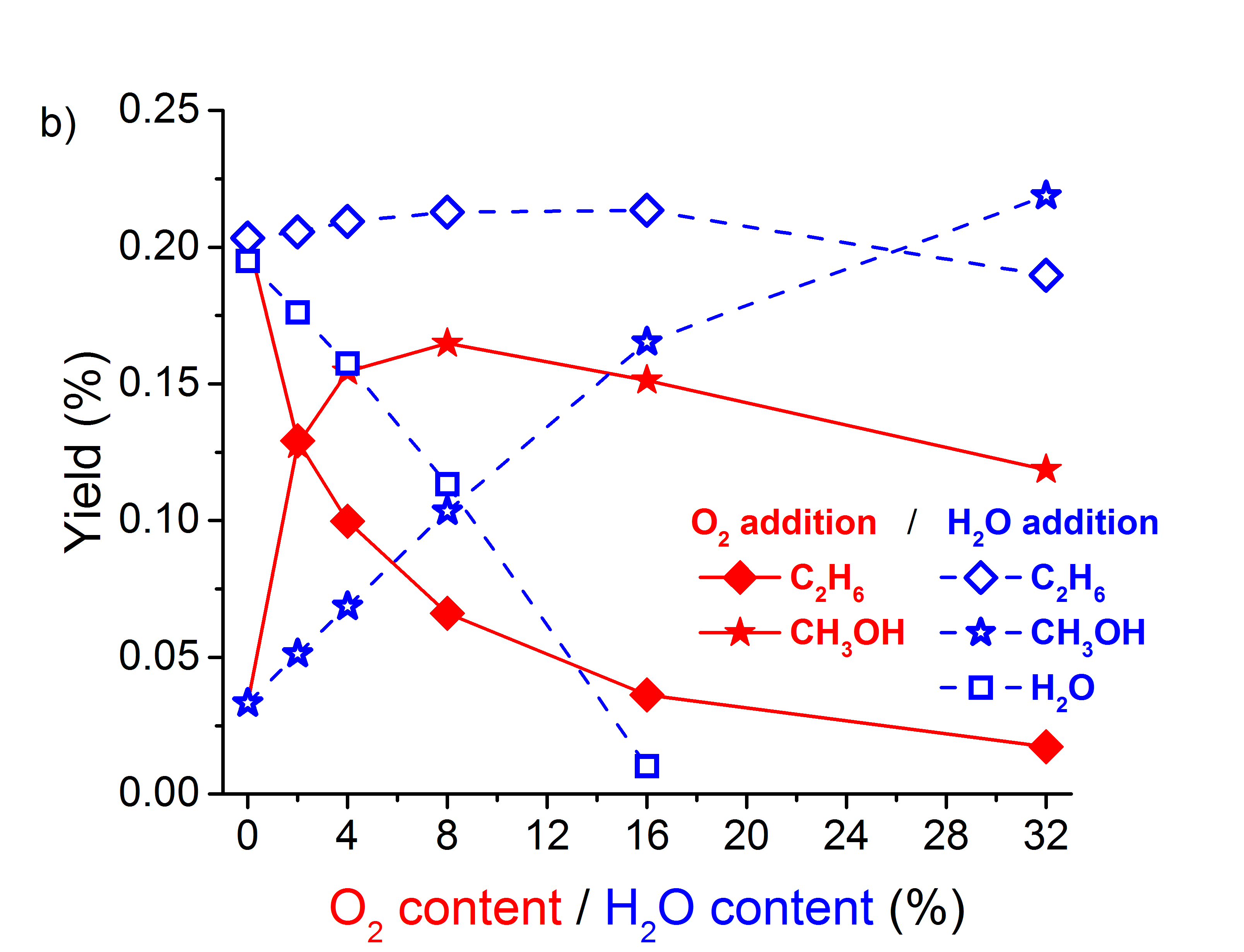


**Figure S3.** Calculated yields towards H_2_O, CO, H_2_, (a) and towards C_2_H_6_ and CH_3_OH (b) as a function of the O_2_ or H_2_O content, for a DBD operating at an SEI of 3 kJ/L, with a mixture of 10 % CH_4_ and 10 % CO_2_ diluted in N_2_.

**3.4. Comparison between experimental and modelling results**

In this section, we discuss the validity of the model by comparing modelling results with experimental data. We provide details on conversions, selectivities, yields and thermal efficiency.

**3.4.1. Conversions**

When O_2_ is added to the DRM mixture (Figure S4a) the CH_4_ conversion increases, while the conversions of CO_2_ and O_2_ decrease. At a low O_2_ concentration_,_ the model underestimates the conversion of O_2_ by 50 %, while at a high O_2_ concentration_,_ it slightly overestimates the conversions of CH_4_ and CO_2_ by 19 and 36 %, respectively. Despite these deviations in absolute values, the increasing conversion of CH_4_ and decreasing conversions of CO_2_ and O_2_ are captured correctly by the model.

The bigger deviation for the conversions of O_2_ and CO_2_ might be attributed to a possible carbon deposition on the surface of the DBD reactor in the experiments. This carbon is then oxidized into CO and CO_2_ by O species. However, the model does not take surface reactions into account, and therefore, this process is neglected. This theory would explain the lower O_2_ conversion and higher CO_2_ conversion, and would be consistent with the fact that our model underestimates the CO selectivity, in comparison with experiments, as discussed in section 3.4.3. below (Figure S6a).

When adding H_2_O to the DRM mixture, the experimental conversions appear to remain rather constant, while the modelling results show a slightly increasing trend. The average deviation for the conversions of CH_4_ and CO_2_ are 12 and 8 %, respectively, indicating that the modelling results are more accurate for the addition of H_2_O than for the addition of O_2_.


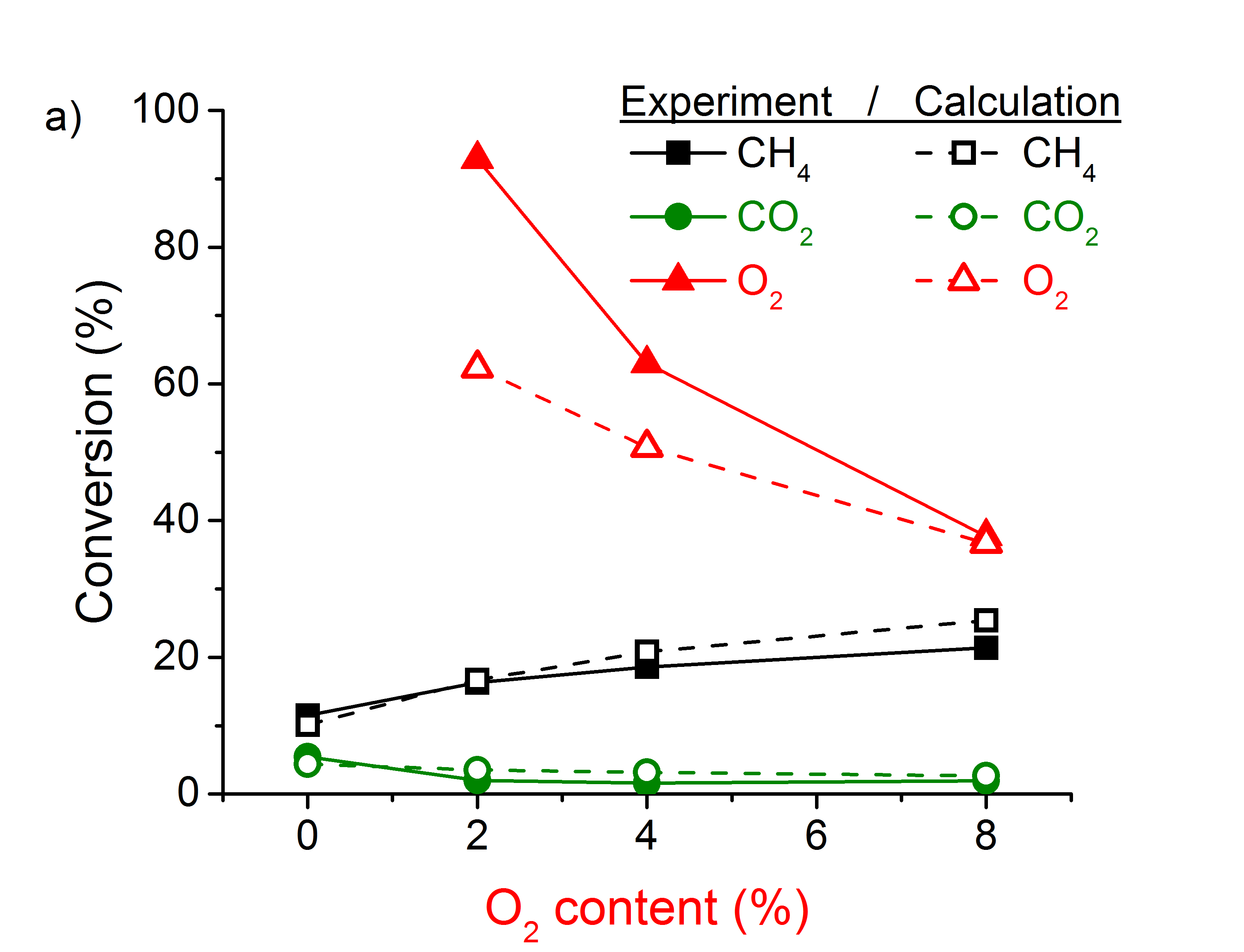

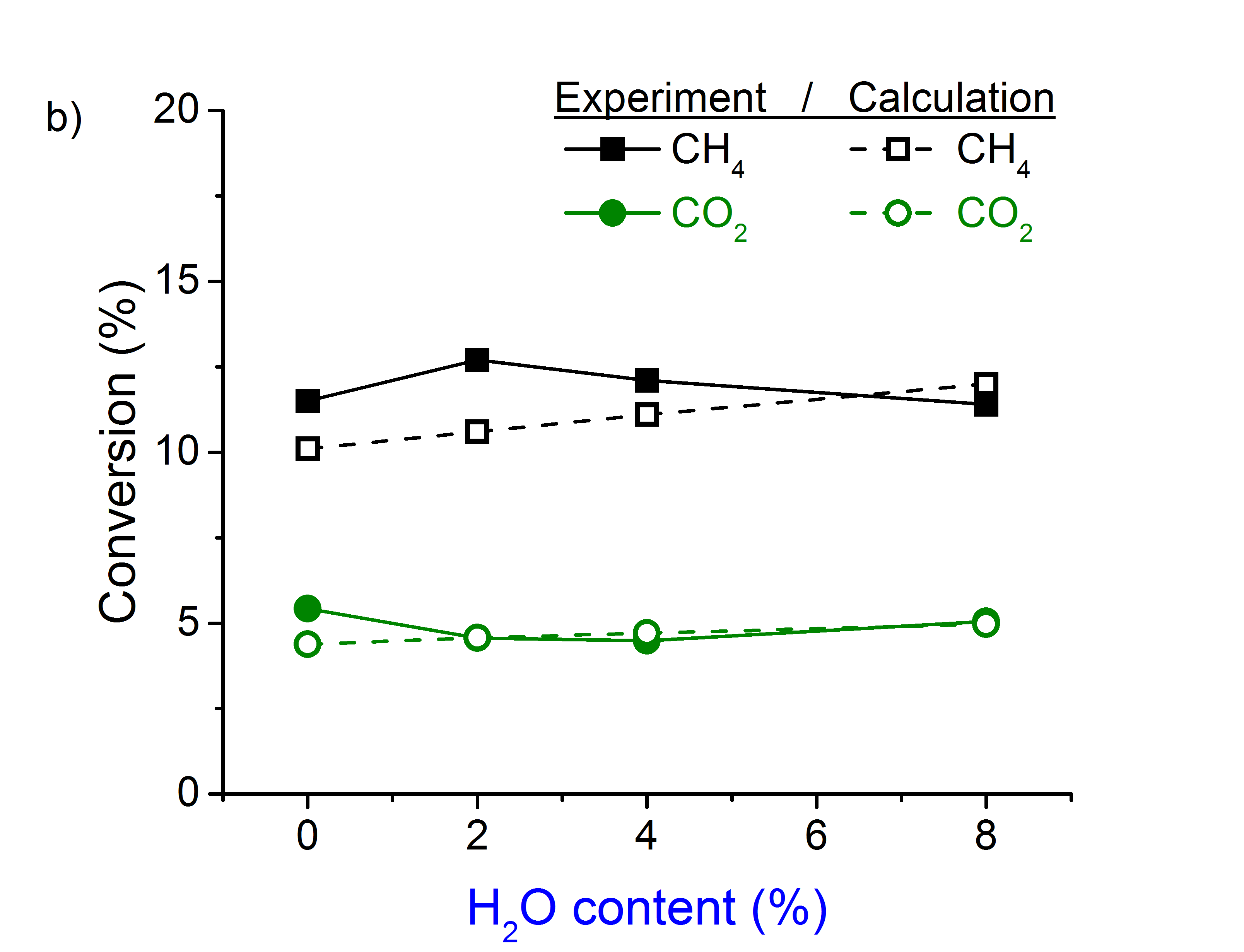


**Figure S4.** Experimental and calculated conversion values for CH_4_, CO_2_ and O_2_ as a function of the O_2_ (a) and H_2_O content (b), for a DBD operating at an SEI of 3 kJ/L, with a mixture of 10 % CH_4_ and 10 % CO_2_ diluted in N_2_.

**3.4.2. Syngas ratio**

Despite some deviations (in absolute values) between the modelling and experimental conversion values, the modelled syngas ratio is in good agreement with the experiments (Figure S5). For the case of pure DRM, we find the syngas ratio to be overestimated by 3 %. When adding 8 % O_2_ the overestimation of the syngas ratio becomes 25 %. When adding 8 % of H_2_O, the syngas ratio is underestimated by 12 %. Both the decreasing trend, when adding O_2_ and increasing trend, when adding H_2_O, are correctly captured by the model.


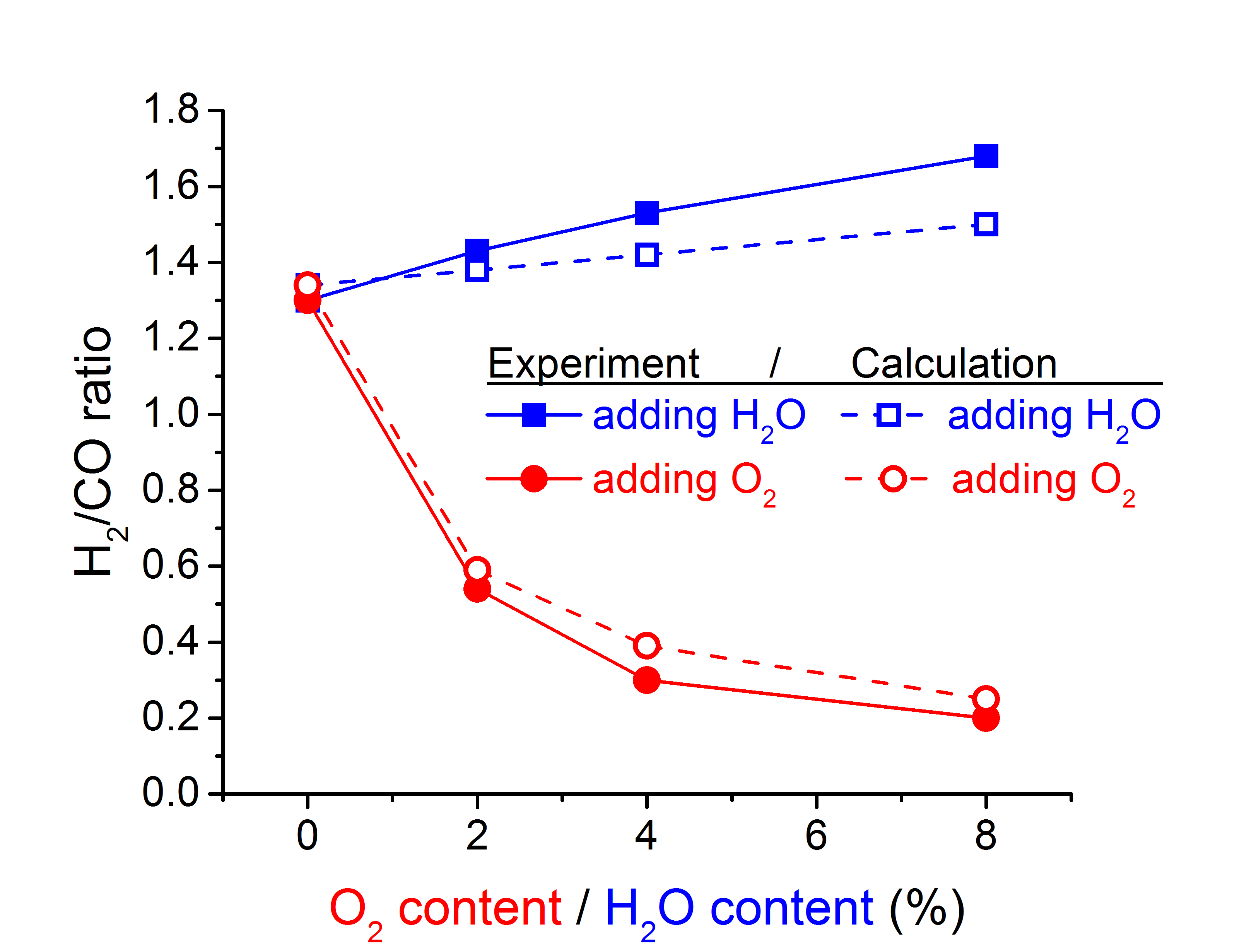


**Figure S5.** Experimental and calculated values of the syngas ratio, as a function of the O_2_ and H_2_O content, for a DBD operating at an SEI of 3 kJ/L, with a mixture of 10 % CH_4_ and 10 % CO_2_ diluted in N_2_.

**3.4.3. Selectivity**

When adding O_2_ to the DRM mixture (Figure S6), the CO selectivity increases at the expense of all the other measured products, i.e., H_2_, C_2_H_6_, C_3_H_8_, C_2_H_4_ and C_2_H_2_. When comparing experimental and modelling results, we find that the model underestimates the selectivity of CO by 25 % on average, upon addition of O_2_. This is obviously closely related to the underestimation of the conversion of O_2_, as previously discussed in section 3.4.1. Vice versa, the model overestimates the selectivity of H_2_ by 15 % on average. The combination of both explains the higher value of syngas ratio obtained in the model, compared to the experimental value. Finally, the hydrocarbon selectivities for C_2_H_6_ and C_3_H_8_, are overestimated, whereas those for C_2_H_4_ and C_2_H_2_ are underestimated. Nevertheless, the model accurately captures the experimental trends for all these different species, upon addition of O_2_.


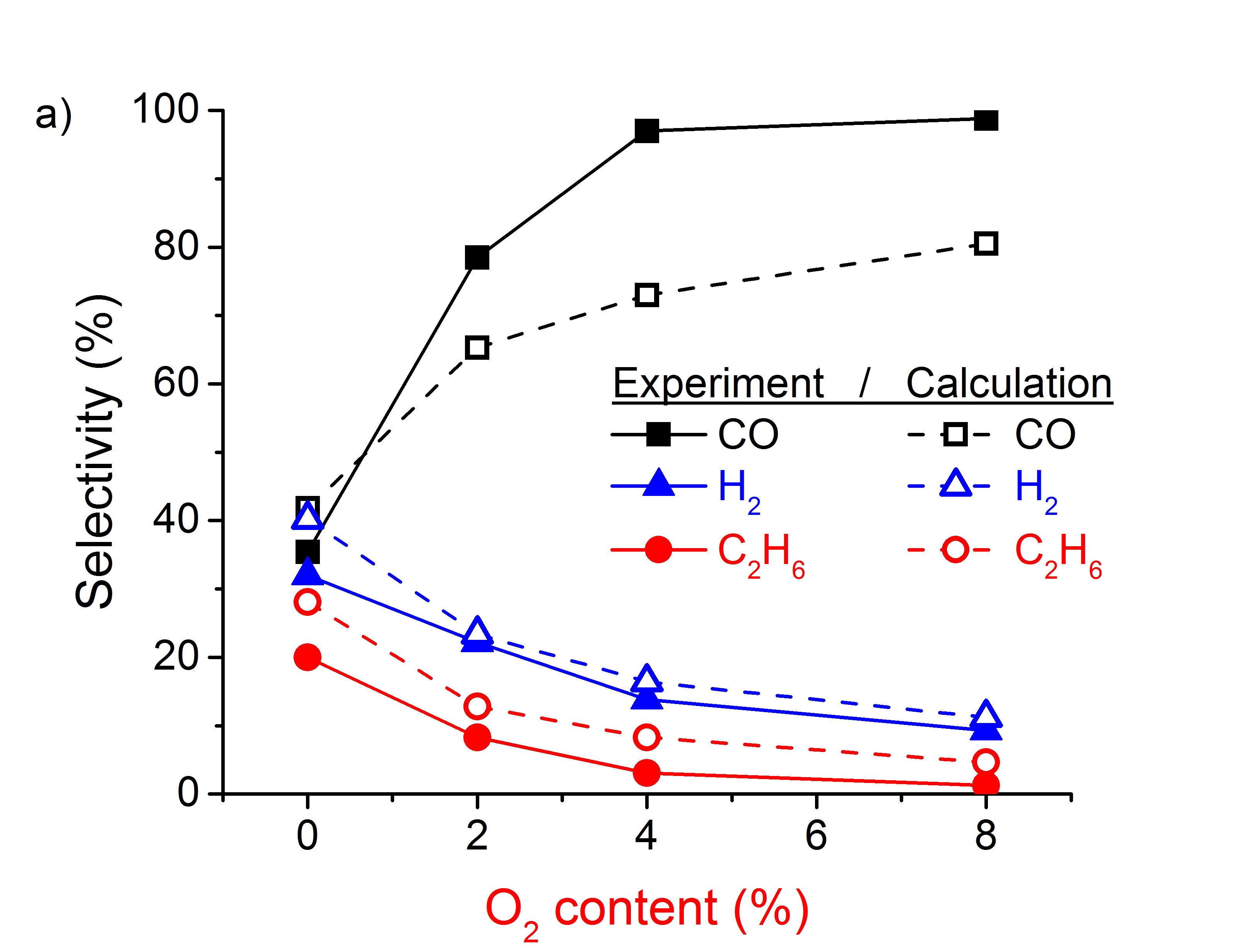

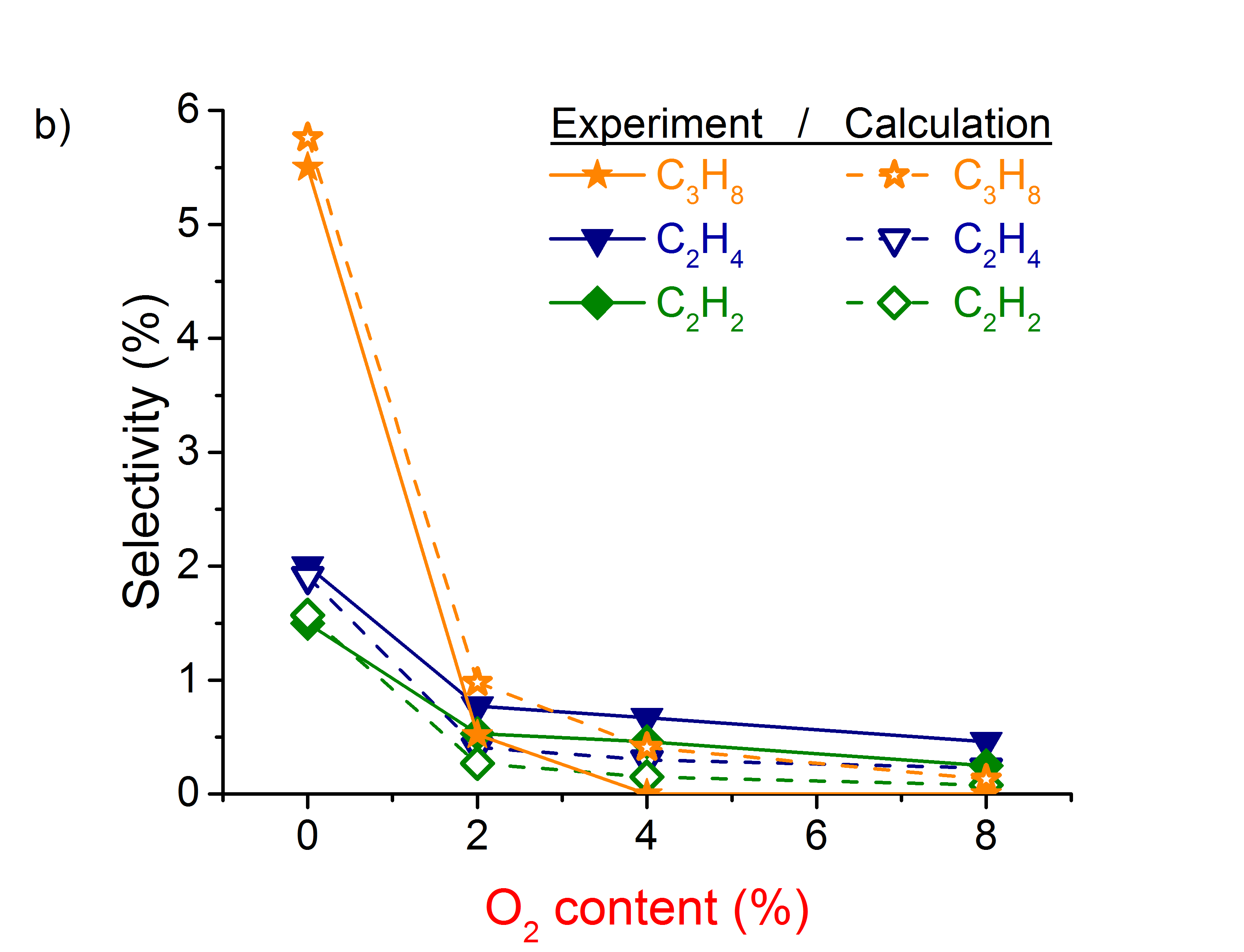


**Figure S6.** Experimental and calculated values of the H-based selectivity towards H_2;_ C-based selectivity towards CO, C_2_H_6_ (a) and C_3_H_8_, C_3_H_6_, C_2_H_4_ and C_2_H_2_ (b), as function of the O_2_ content, for a DBD operating at an SEI of 3 kJ/L, with a mixture of 10 % CH_4_ and 10 % CO_2_ diluted in N_2_.

When adding H_2_O to the DRM mixture (Figure S7), the selectivities of H_2_ and CO increase slowly, while the hydrocarbon selectivities decrease slightly. When comparing experimental and modelling results, we find that the model overestimates the selectivities of H_2_ and CO by 12 % and by 10 % on average, respectively. The selectivity is slightly overestimated for all hydrocarbons, C_2_H_6_, C_3_H_8_, C_2_H_4_ and C_2_H_2_, by an average of 25, 4, 1 and 10 %, respectively. For the addition of H_2_O, the model also accurately captures the experimental trends for all these different species.


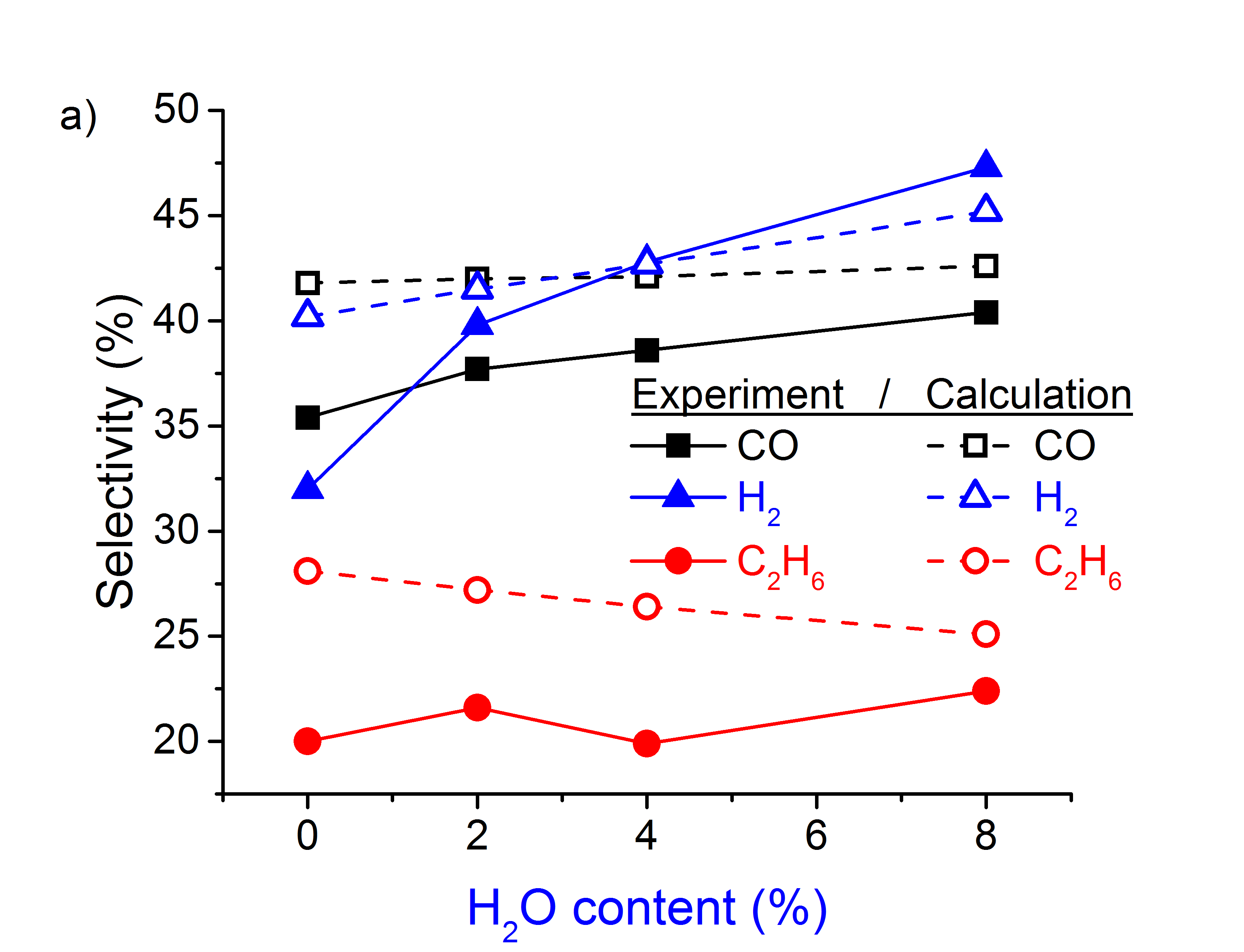

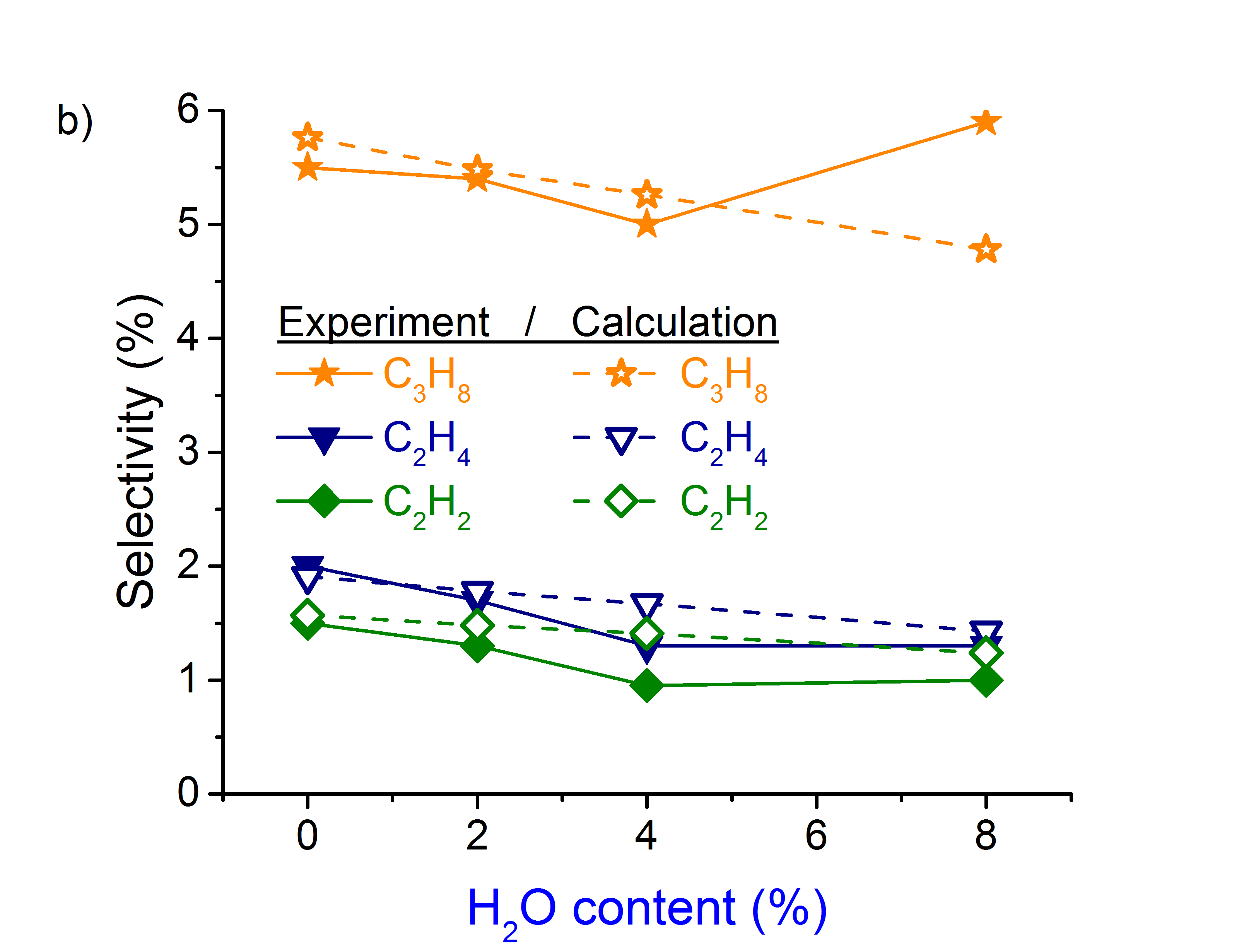


**Figure S7.** Experimental and calculated values of H-based selectivity towards H_2,_ C-based selectivity towards CO, C_2_H_6_ (a), C_3_H_8_, C_3_H_6_, C_2_H_4_ and C_2_H_2_ (b), as function of the H_2_O content, for a DBD operating at an SEI of 3 kJ/L, with a mixture of 10 % CH_4_ and 10 % CO_2_ diluted in N_2_.

**3.4.4. Yield**

Since the yield is proportional to the product of the effective conversion and the selectivity, we observe almost the same trends for the conversion and selectivity as we do for the yields. Additionally, when comparing the experimental and modelling results, we expect to obtain the same accumulation of the deviations as that observed for the conversion (Section 3.4.1.) and selectivity (Section 3.4.3.).

For the addition of O_2_ (Figure S8), the accumulation of deviations is the most visible for the CO yield. Despite the underestimation of the CO selectivity (Figure S6), the overestimation in the CO_2_ conversion (Figure S4) causes the CO yield to be almost identical upon addition of 4 % O_2_, and to be overestimated for 8 % of added O_2_. In comparison, the overestimation of the H_2_ selectivity and CH_4_ conversion leads to a bigger overestimation of the H_2_ yield. Besides the C_2_H_6_ yield, all hydrocarbon yields are underestimated. However, since the model correctly captures both the experimental conversion and the trends in selectivity, the model also accurately captures the trends in experimental yield.


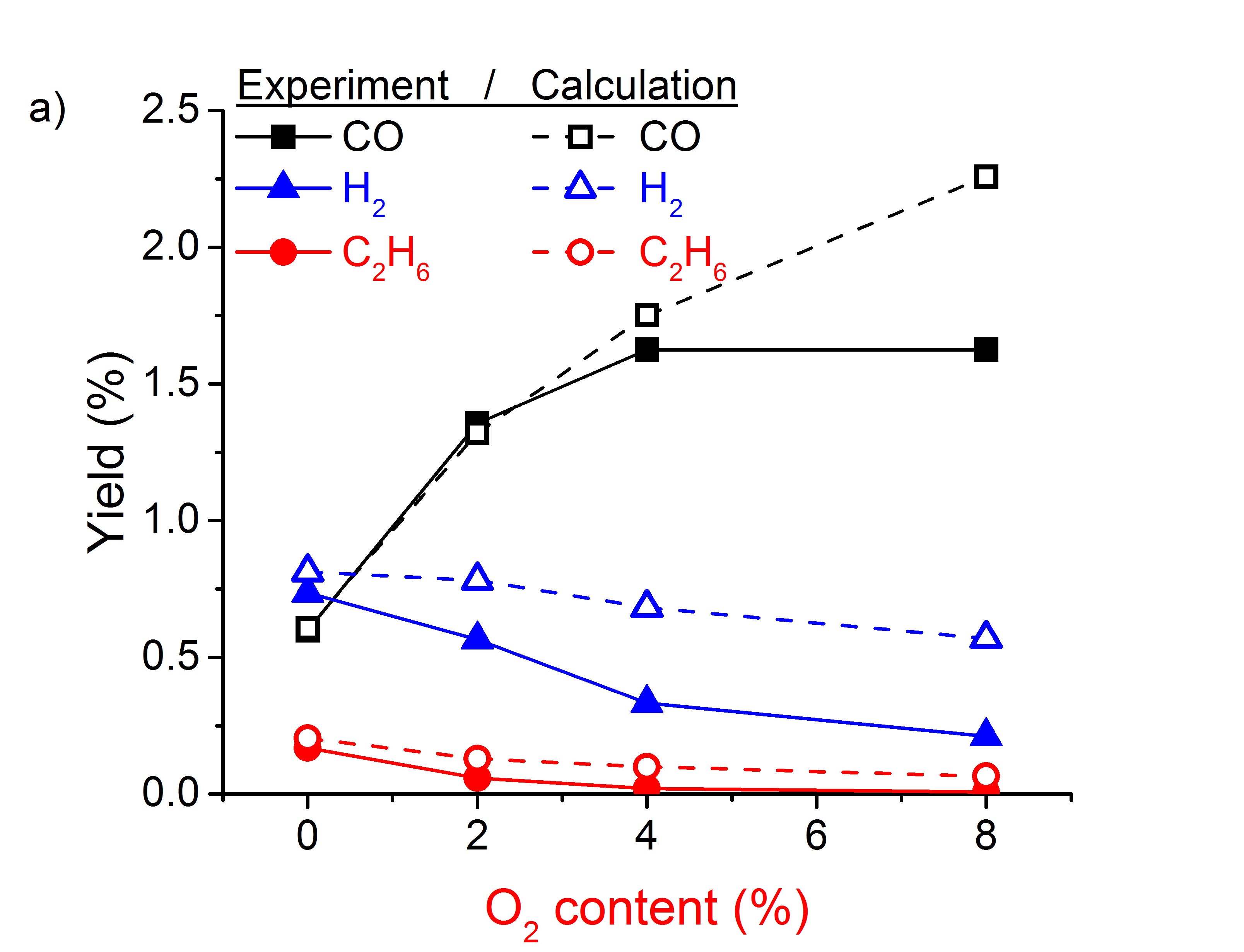

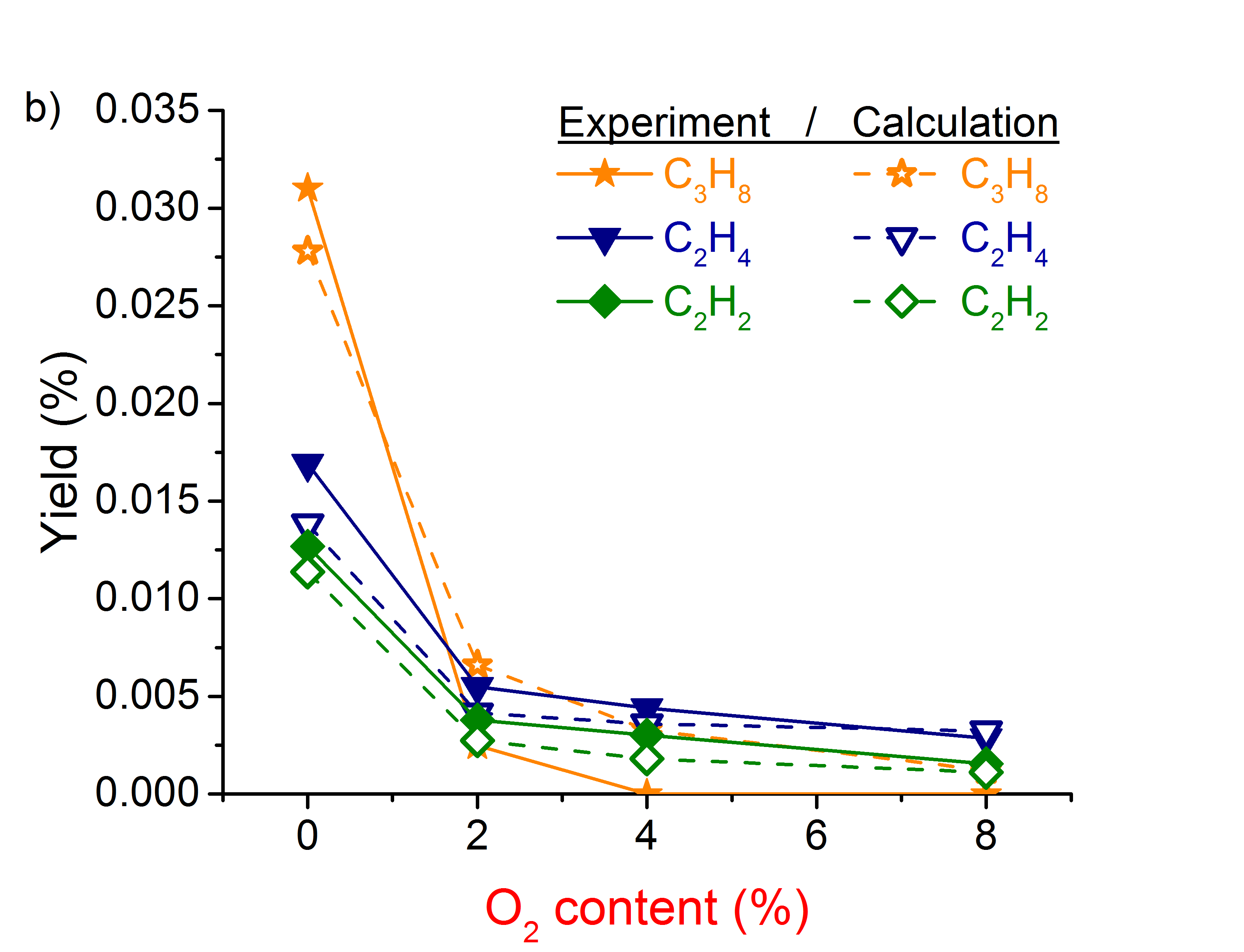


**Figure S8.** Experimental and calculated yields towards H_2_, CO, C_2_H_6_ (a) and towards C_3_H_8_, C_3_H_6_, C_2_H_4_ and C_2_H_2_ (b), as function of the O_2_ content, for a DBD operating at an SEI of 3 kJ/L, with a mixture of 10 % CH_4_ and 10 % CO_2_ diluted in N_2_.

For the addition of H_2_O (Figure S9), the deviations on the conversions and selectivities are much smaller than for the addition of O_2_, and as a result, the deviations on the yields are comparable. Since the model correctly captures both the experimental conversion and the trends in selectivity, it appears logical that it also accurately captures the trends in experimental yield.


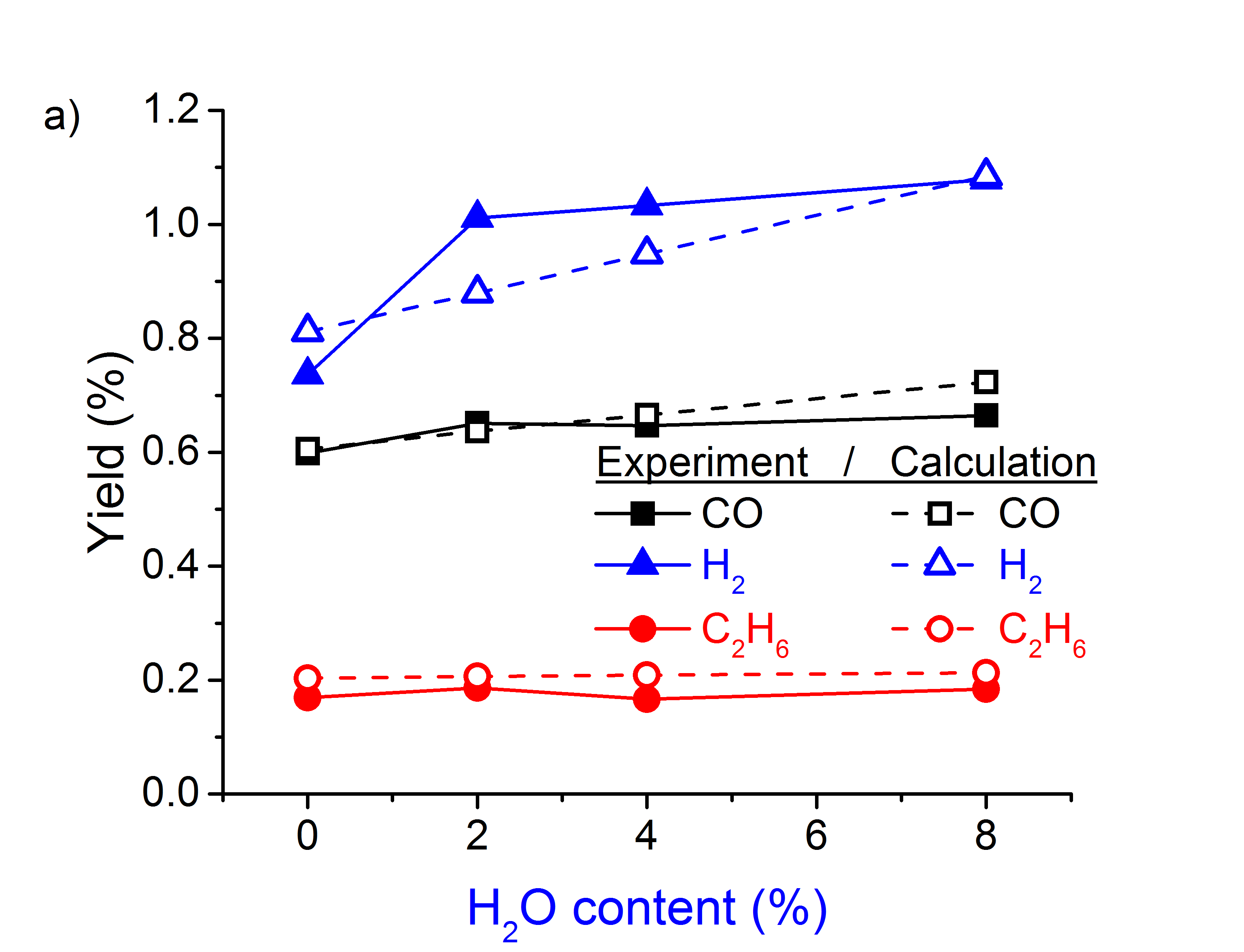

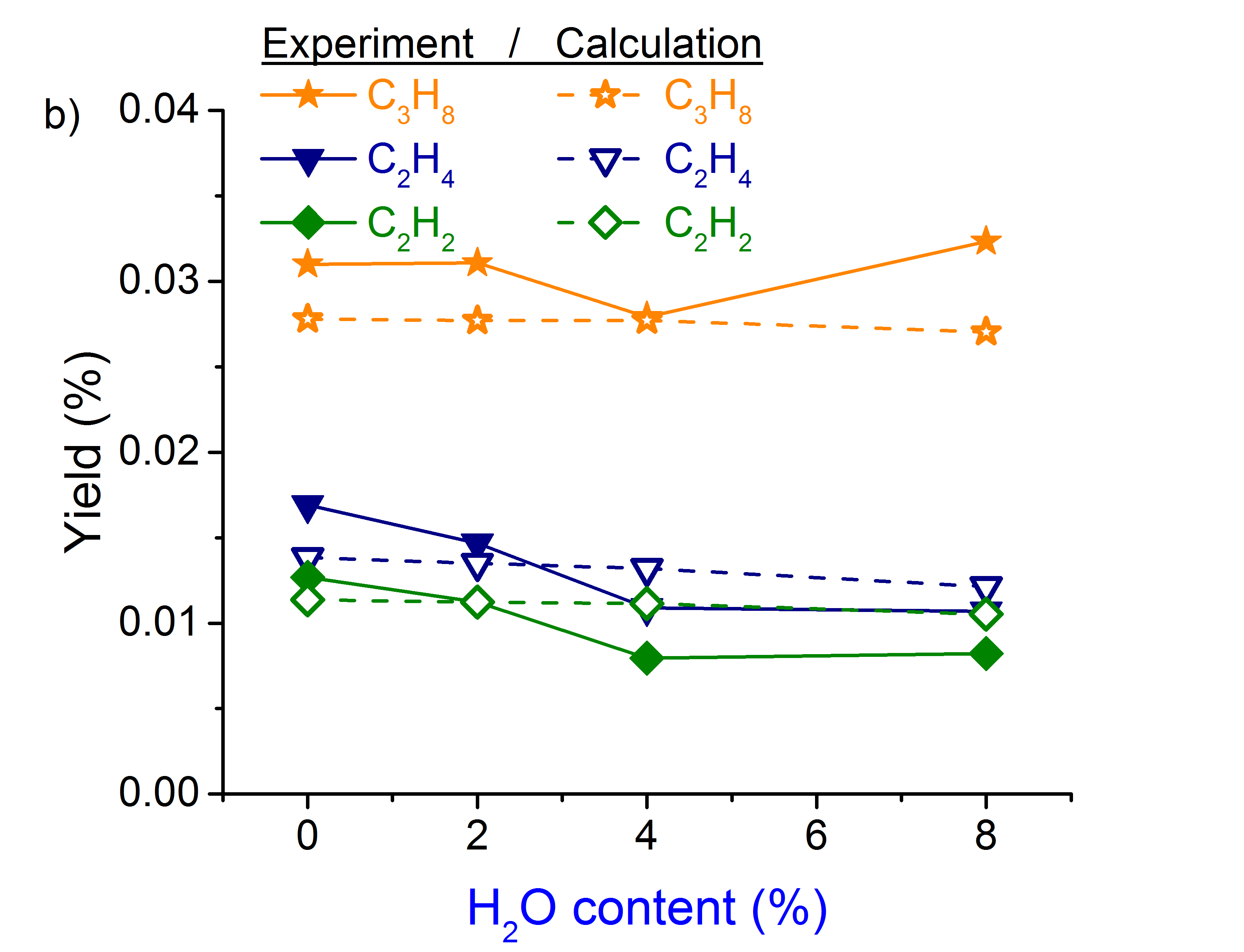


**Figure S9.** Experimental and calculated yields towards H_2_, CO, C_2_H_6_ (a) and towards C_3_H_8_, C_3_H_6_, C_2_H_4_ and C_2_H_2_ (b), as function of the H_2_O content, for a DBD operating at an SEI of 3 kJ/L, with a mixture of 10 % CH_4_ and 10 % CO_2_ diluted in N_2_.

**3.4.5. Thermal efficiency**

The thermal efficiency for the experimental results is determined based on the following measured products: H_2_, CO, C_3_H_8_, C_2_H_6_, C_2_H_4_ and C_2_H_2_. For the modelling results, the thermal efficiency is calculated for the two following cases: (1) we use the same products as those taken into account for the experimental results, and (2) we consider the products used in (1) with the addition of methanol (CH_3_OH), which we were not able to measure experimentally with the current experimental set-up.

When comparing the modelling results from those two cases, we find that the thermal efficiency is about 5–10 % higher with addition of methanol (Figure S10). When comparing the experimental results with the modelling results that do not contain methanol, we notice that the model overestimates the thermal efficiency by approximately 5–10 % for both the additions of O_2_ and H_2_O However, most importantly, the trends are again captured correctly by the model.

**
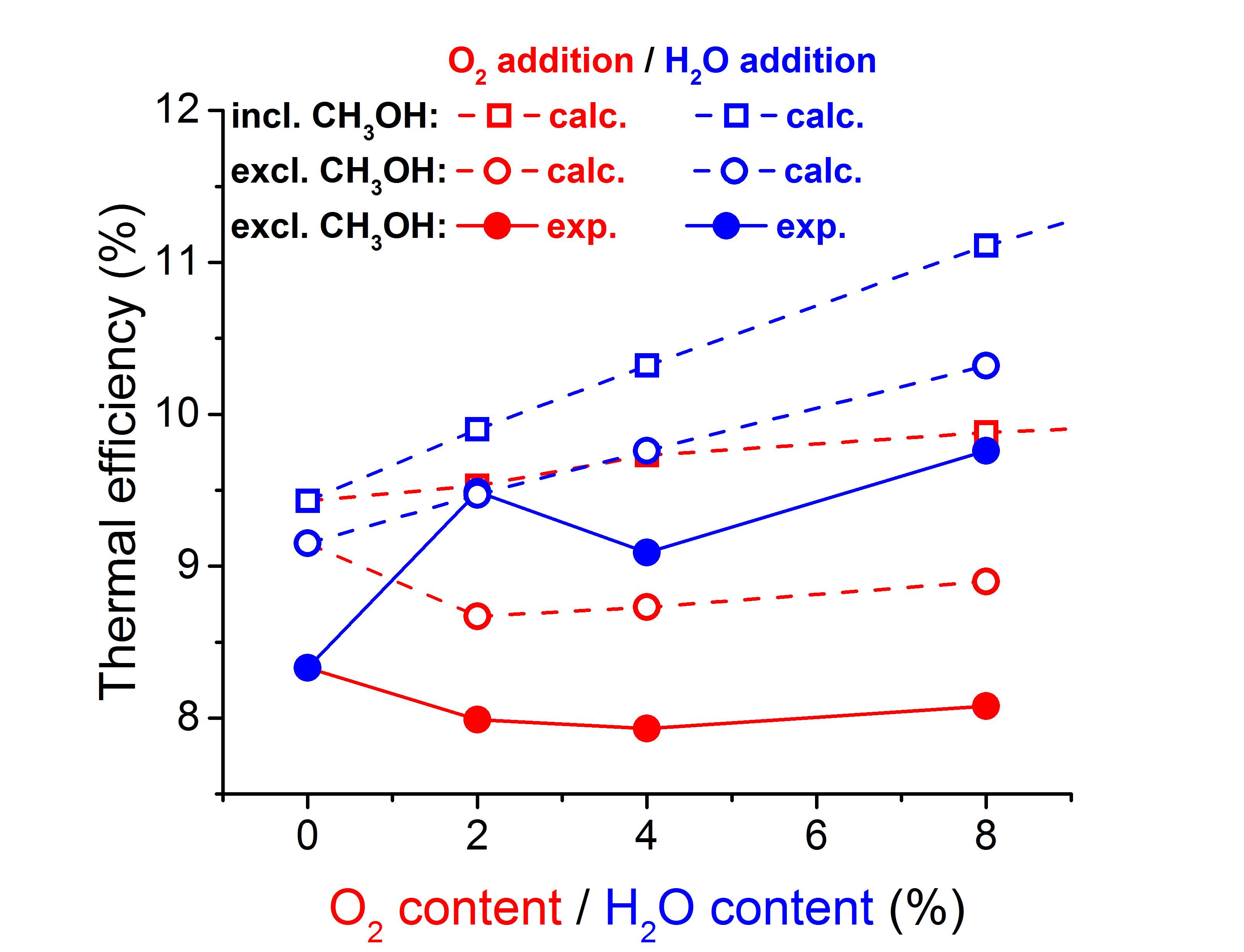
**

**Figure S10.** Experimental and calculated thermal efficiencies as function of the O_2_ and H_2_O content, for a DBD operating at an SEI of 3 kJ/L, with a mixture of 10 % CH_4_ and 10 % CO_2_ diluted in N_2_.

**3.4.6. Conclusion**

From the comparison between the calculations and experiments made for the conversions, selectivities, yields and thermal efficiency, we conclude that the model is capable of accurately capturing the different trends observed experimentally. When looking at the absolute values, there are some deviations (generally 10–20 %) from the experimental data, but the trends upon addition of O_2_ or H_2_O are captured accurately. Therefore, the chemistry set used in this model is able to capture the underlying plasma chemistry and the chemical pathways to the formed products, and thus, we are confident that this model can be used to describe the trends of the plasma-based multi-reforming process.

Additionally, we are confident that the trends in methanol yield discussed here are accurate and demonstrate the potential of plasma-based multi-reforming, for two main reasons. (1) Although our current equipment cannot measure methanol, the model is capable of capturing the underlying plasma chemistry and chemical pathways to all the other validated main products. (2) When we examine closely the experimental carbon and hydrogen balances derived from the measured products (as presented in the tables in Section 3.1. and 3.2. above), we notice that, upon addition of O_2_, the carbon balance reaches 100 %, whereas the hydrogen balance decrease steadily. These trends clearly indicate the formation of water, as shown by the modelling results. Upon addition of H_2_O, on the other hand, the hydrogen balance increases from 60 to 80 % (which is related to the decrease in H_2_O formation), but the carbon balance remains approximately 70 %. Additionally, if we were to construct the oxygen balance, around 30–45 % of the oxygen would be missing. This indicates that 30 % of the converted carbon, 20–40 % of the converted hydrogen and 30–45 % of the converted oxygen have to be captured in the liquid products.

**4. References**

1. Zhang, X. & Cha, M. S. Electron-induced dry reforming of methane in a temperature-controlled dielectric barrier discharge reactor. *J. Phys. D. Appl. Phys.* **46,** 415205 (2013).

2. Zhang, X. & Cha, M. S. Partial oxidation of methane in a temperature-controlled dielectric barrier discharge reactor. *Proc. Combust. Inst.* **35,** 3447–3454 (2015).

3. Liu, J.-L., Snoeckx, R. & Cha, M. S. Steam reforming of methane in a temperature-controlled dielectric barrier discharge reactor: the role of electron-induced chemistry versus thermochemistry. *J. Phys. D. Appl. Phys.* **51,** 385201 (2018).

4. Pinhão, N., Moura, A., Branco, J. B. & Neves, J. Influence of gas expansion on process parameters in non-thermal plasma plug-flow reactors: A study applied to dry reforming of methane. *Int. J. Hydrogen Energy* **41,** 9245–9255 (2016).

5. Snoeckx, R. & Bogaerts, A. Plasma technology – a novel solution for CO2 conversion? *Chem. Soc. Rev.* **46,** 5805–5863 (2017).

6. Snoeckx, R., Rabinovich, A., Dobrynin, D., Bogaerts, A. & Fridman, A. Plasma-based liquefaction of methane: The road from hydrogen production to direct methane liquefaction. *Plasma Process. Polym.* **14,** 1600115 (2017).

7. Pancheshnyi, S., Eismann, B., Hagelaar, G. J. M. & Pitchford, L. C. Computer code ZDPlasKin. (2008).

8. Hagelaar, G. J. M. & Pitchford, L. C. Solving the Boltzmann equation to obtain electron transport coefficients and rate coefficients for fluid models. *Plasma Sources Sci. Technol.* **14,** 722–733 (2005).

9. Wang, W., Snoeckx, R., Zhang, X., Cha, M. S. & Bogaerts, A. Modeling Plasma-based CO2 and CH4 Conversion in Mixtures with N2 , O2 , and H2O: The Bigger Plasma Chemistry Picture. *J. Phys. Chem. C* **122,** 8704–8723 (2018).

10. Wang, W., Berthelot, A., Zhang, Q. & Bogaerts, A. Modelling of plasma-based dry reforming: How do uncertainties in the input data affect the calculation results? *J. Phys. D. Appl. Phys.* **51,** (2018).

11. Bogaerts, A., Wang, W., Berthelot, A. & Guerra, V. Modeling plasma-based CO2 conversion: crucial role of the dissociation cross section. *Plasma Sources Sci. Technol.* **25,** 055016 (2016).

12. Aerts, R., Somers, W. & Bogaerts, A. Carbon Dioxide Splitting in a Dielectric Barrier Discharge Plasma: A Combined Experimental and Computational Study. *ChemSusChem* **8,** 702–716 (2015).

13. Snoeckx, R., Aerts, R., Tu, X. & Bogaerts, A. Plasma-Based Dry Reforming: A Computational Study Ranging from the Nanoseconds to Seconds Time Scale. *J. Phys. Chem. C* **117,** 4957–4970 (2013).

14. Snoeckx, R., Heijkers, S., Van Wesenbeeck, K., Lenaerts, S. & Bogaerts, A. CO 2 conversion in a dielectric barrier discharge plasma: N 2 in the mix as a helping hand or problematic impurity? *Energy Environ. Sci.* **9,** 999–1011 (2016).

15. Snoeckx, R., Ozkan, A., Reniers, F. & Bogaerts, A. The Quest for Value-Added Products from Carbon Dioxide and Water in a Dielectric Barrier Discharge: A Chemical Kinetics Study. *ChemSusChem* **10,** 409–424 (2017).

16. Snoeckx, R. *et al.* Influence of N2 concentration in a CH4/N2 dielectric barrier discharge used for CH4 conversion into H2. *Int. J. Hydrogen Energy* **38,** 16098–16120 (2013).

17. Snoeckx, R., Zeng, Y. X., Tu, X. & Bogaerts, A. Plasma-based dry reforming: improving the conversion and energy efficiency in a dielectric barrier discharge. *RSC Adv.* **5,** 29799–29808 (2015).

18. Aerts, R., Snoeckx, R. & Bogaerts, A. In-Situ Chemical Trapping of Oxygen in the Splitting of Carbon Dioxide by Plasma. *Plasma Process. Polym.* **11,** 985–992 (2014).
